# Heat shock protein A1L restricts influenza A virus by ubiquitination of NA

**DOI:** 10.1128/jvi.00771-25

**Published:** 2025-08-11

**Authors:** Yan Yan, Jianan Xu, Zhen Chen, Yuting Xu, Linlong Qin, Lingyan Zhao, Hongli Zhang, Xiaoxiao Feng, Chaoliang Yao, Yu Huang, Jiyong Zhou, Tingjuan Deng

**Affiliations:** 1 MOA Key Laboratory of Animal Virology, Zhejiang University Center for Veterinary Sciences, Hangzhou, China; 2 State Key Laboratory for Diagnosis and Treatment of Severe Infectious Diseases, First Affiliated Hospital, Zhejiang University 12377, Hangzhou, China; 3 Institute of Animal Husbandry and Veterinary, Fujian Academy of Agricultural Sciences 660874 , Fuzhou, China; 4 Zhejiang Provincial Center for Animal Disease Control and Prevention, Hangzhou, China; University Medical Center Freiburg, Freiburg, Germany

**Keywords:** influenza A virus, neuraminidase, heat shock protein A1L, ubiquitination, viral replication

## Abstract

Neuraminidase (NA) protein of influenza A virus (IAV), a tetrameric envelope glycoprotein critical for viral release and spread, has well-characterized enzymatic functions but poorly resolved host-pathogen interactions. However, host factors targeted by NA or those regulating NA activity are largely uncharacterized. Here, we identified host heat shock protein A1L (HSPA1L) as a novel NA-binding partner that suppresses IAV replication. Subsequent studies revealed that HSPA1L promotes NA ubiquitination at K242, resulting in its NBR1-dependent autophagic degradation. Moreover, a recombinant H5N1 virus carrying the NA^K242R^ mutation exhibited enhanced replication and resistance to HSPA1L-mediated viral suppression compared to the wild-type (WT) virus. Collectively, we unveil an intrinsic defense mechanism, wherein HSPA1L antagonizes IAV by promoting NBR1-dependent autophagic degradation of NA via site-specific ubiquitination.

**IMPORTANCE:**

IAV, especially avian influenza virus H5N1, begins to cause the infection and/or death for its new uncommon host, especially sea mammals and ruminants, indicating that the virus is adapting to mammalian infections. These developments substantially elevate concerns about pandemic potential and zoonotic risk escalation, necessitating a deeper understanding of host-IAV interactions to develop effective antiviral countermeasures. HSP70 family proteins are known to modulate viral infections. However, the specific role of HSPA1L, a member of the HSP70 family, in IAV infection remains poorly characterized. In this study, we demonstrate that HSPA1L defends against IAV by regulating NA proteostasis. Mechanistically, HSPA1L directly binds NA, promotes its ubiquitination at K242, and mediates NBR1-dependent autophagic degradation, thereby suppressing viral replication. Our study identifies HSPA1L as a promising target for antiviral strategies against IAV.

## INTRODUCTION

Influenza A virus is a human respiratory pathogen that causes seasonal epidemics and occasional global pandemics associated with significant morbidity and mortality (1). In recent years, IAV have demonstrated a notable expansion in their host range, raising significant public health concerns. Of particular note is the highly pathogenic avian influenza A virus H5N1, a subtype of IAVs, which has been increasingly reported in various mammals (including skunks, foxes, bears, minks, seals, porpoises, sea lions, and dolphins) from Asia, America, and Europe (2). Since March 2024, an unprecedented outbreak of H5N1 virus infection in cattle herds occurred in the USA, characterized by viral spread within and between herds, infections in poultry and domestic cats, and spillover events into humans, collectively indicating an increased public health risk (3, 4). Therefore, under the ongoing global outbreaks of H5N1 virus infection, it is very necessary to understand the underlying mechanism of host-H5N1 virus interaction.

Influenza A virus belongs to the *Orthomyxoviridae* family and is characterized by its hemagglutinin (H5) and neuraminidase (N1) surface proteins (5). The genome is composed of single-stranded negative-sense RNA segments, encoding at least 11 proteins (6–8). Among these proteins, its surface glycoprotein NA has garnered attention due to its critical role in the influenza virus life cycle, particularly in facilitating the release of new virions from infected cells and thus promoting viral spread (9–14). The function of NA is indispensable not only for the replication of the virus but also for its pathogenicity and transmissibility, making it a key target for therapeutic interventions and vaccine development (15). The amino acid sequence of NA is highly conserved among various influenza virus strains, which contributes to its functional stability and enzymatic activity (16). However, little is understood about the natural defenses employed by host cells to defend against the IAV NA.

Heat shock proteins (HSPs) are a group of highly conserved proteins that play crucial roles in cellular stress responses, protein folding, and the maintenance of proteostasis (17). HSPs are classified based on their molecular weight and structural characteristics into several families, each playing distinct roles in cellular processes (18). The HSP70 family is one of the most conserved protein families (19). HSP70 proteins consist of two major functional domains: an N-terminal nucleotide-binding domain with ATPase activity, and a C-terminal substrate binding domain (20–25). Their involvement in viral infections has garnered significant attention in recent years, as they can act both as facilitators and inhibitors of viral propagation. Senecavirus A, Enterovirus 71, Zika virus, cucumber necrosis virus, and porcine reproductive and respiratory syndrome virus interact with or recruit HSP70 through their viral proteins which enhanced viral infection (26–30). Conversely, HSP70 exhibits antiviral responses against certain viruses. For example, HSP70 interacted with viral Hexon protein for degradation, leading to inhibition of Fowl adenovirus serotype 4 replication (31), and the ability of IAV polymerase to bind to viral RNA was blocked by HSP70 (32) and HSP70 induced type I interferon production, thereby inhibiting measles virus replication (33). HSP70 participates in multiple stages of the viral life cycle, including attachment (28), entry (34, 35), replication (34, 36), and assemblage (37). HSPA1L is a constitutively expressed, non-inducible cytosolic protein with high abundance in testis (38, 39). However, the role of HSPA1L in viral infection remains enigmatic.

The objective of this study was to explore whether HSP70 family proteins modulate IAV infection by targeting NA protein and to elucidate the underlying regulatory mechanism. Mass spectrometry analysis of H5N1-NA protein complexes detected peptide sequences unique to HSPA1L, with no other HSP70 family members identified. Further studies confirmed that HSPA1L is a novel host interactor that directly binds NA proteins and restricts IAV replication. Mechanistically, HSPA1L promotes NA ubiquitination and subsequent NBR1-dependent autophagic degradation, uncovering its role in intrinsic antiviral defense.

## RESULTS

### HSPA1L interacts with NA protein

**Fig 1:**
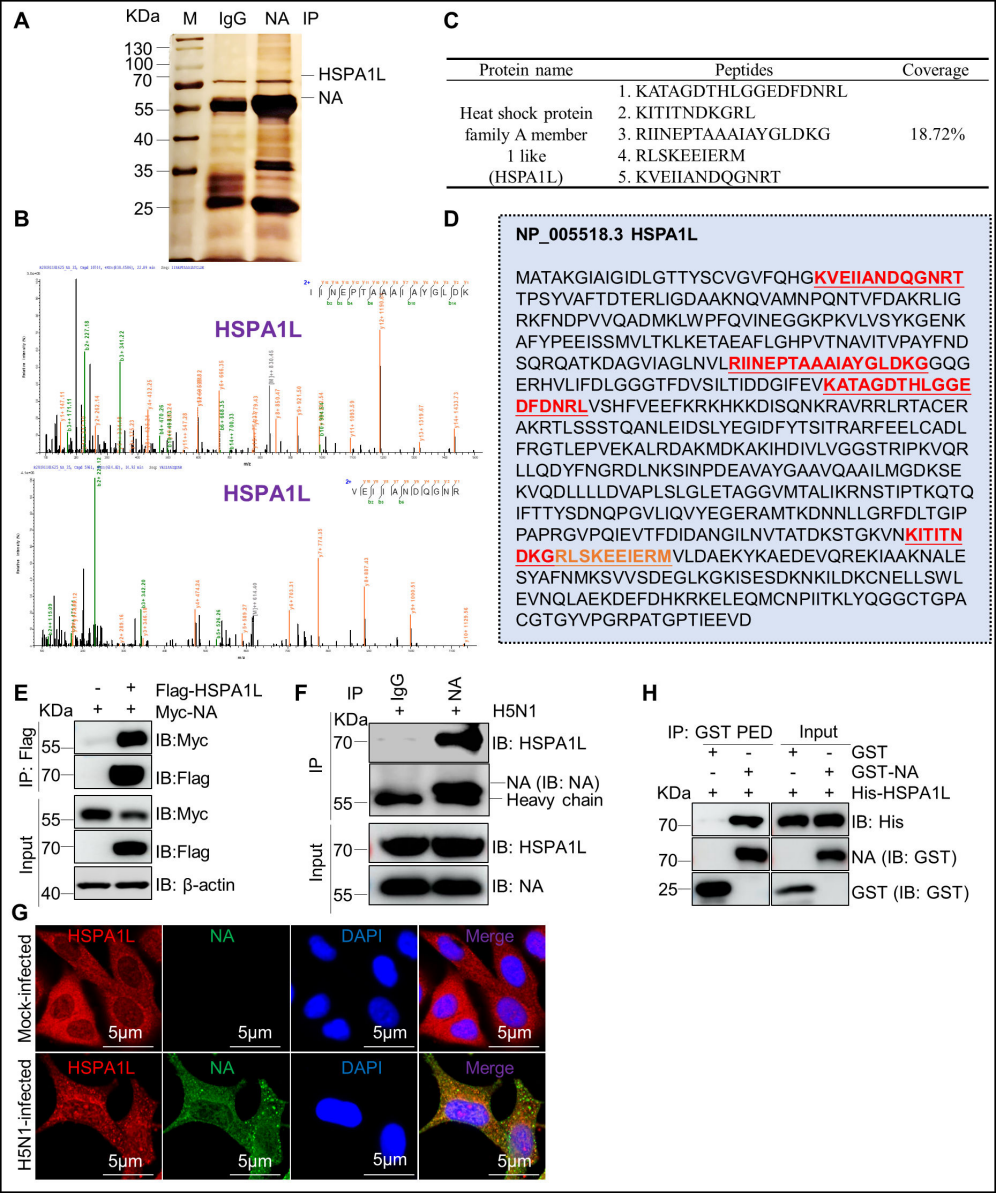
Identification of HSPA1L as a binding partner of NA. (**A**) A549 cells were infected with H5N1 (MOI = 3) for 18 h. The resultant cells were collected and subjected to co-immunoprecipitation assays using mouse anti-NA monoclonal antibody (mAb) or a control IgG. The immunoprecipitated complexes were subsequently separated by SDS-PAGE and visualized through silver staining. Then, the silver staining gels were manually excised and subjected to mass spectrometry analysis. This analysis led to the identification of HSPA1L as a potential interacting protein. (**B**) The MS spectra of the selected HSPA1L peptides, IINEPTAAAIAYGLDK and VEIIANDQGNRT, are shown. (**C**) Coverage of identified peptides in the complete length of HSPA1L. (**D**) Identified peptides located in the HSPA1L sequence. (**E**) HSPA1L interacts with NA during transfection. HEK293T cells were co-transfected with Flag-HSPA1L and Myc-NA for 36 h. The cellular lysates were subjected to Flag-immunoprecipitation and western blotting with indicated antibodies. (**F**) Endogenous HSPA1L associates with NA during H5N1 infection. A549 cells were infected with H5N1 (MOI = 3) for 18 h. The lysates were subjected to co-immunoprecipitation with anti-NA mouse mAb and western blotting assay using indicated antibodies. (**G**) Analysis of NA and HSPA1L colocalization in H5N1-infected A549 cells. Confocal assays were performed as described in Materials and Methods. Scale bar, 5 µm. (**H**) The interaction of NA and HSPA1L was analyzed by GST pull-down assay.

The HSP70 family of proteins has been implicated in regulating multiple stages of viral infection, including viral attachment (28), entry (34, 35), replication (34, 36), and assembly (37). However, their roles in IAV infections remain largely uncharacterized. The surface viral protein NA is crucial for viral replication, pathogenicity, and transmissibility (15). Therefore, we hypothesize that HSP70 family proteins may target NA to modulate IAV infections. To test this hypothesis, we generated and purified a mouse monoclonal antibody against the H5N1-NA protein and subsequently used it for co-immunoprecipitation (Co-IP) assays. Immunoprecipitated NA complexes isolated from H5N1-infected A549 cells were analyzed by mass spectrometry. As demonstrated in Fig. 1A and B, HSPA1L is a putative interactor of H5N1-NA. Mass spectrometry analysis identified five unique peptides corresponding to HSPA1L, achieving 18.72% sequence coverage (Fig. 1C and D). Subsequently, an HSPA1L expression vector was constructed and co-transfected with Myc-H5N1-NA into HEK293T cells. Co-IP assays confirmed the physical interaction between HSPA1L and H5N1-NA (Fig. 1E). Furthermore, endogenous HSPA1L co-precipitated and colocalized with the NA protein in H5N1-infected A549 cells (Fig. 1F and G). Glutathione S-transferase (GST)-pulldown assays further demonstrated a direct interaction between HSPA1L and NA (Fig. 1H). Taken together, these findings establish HSPA1L as a specific binding partner of H5N1-NA.

**Fig 2:**
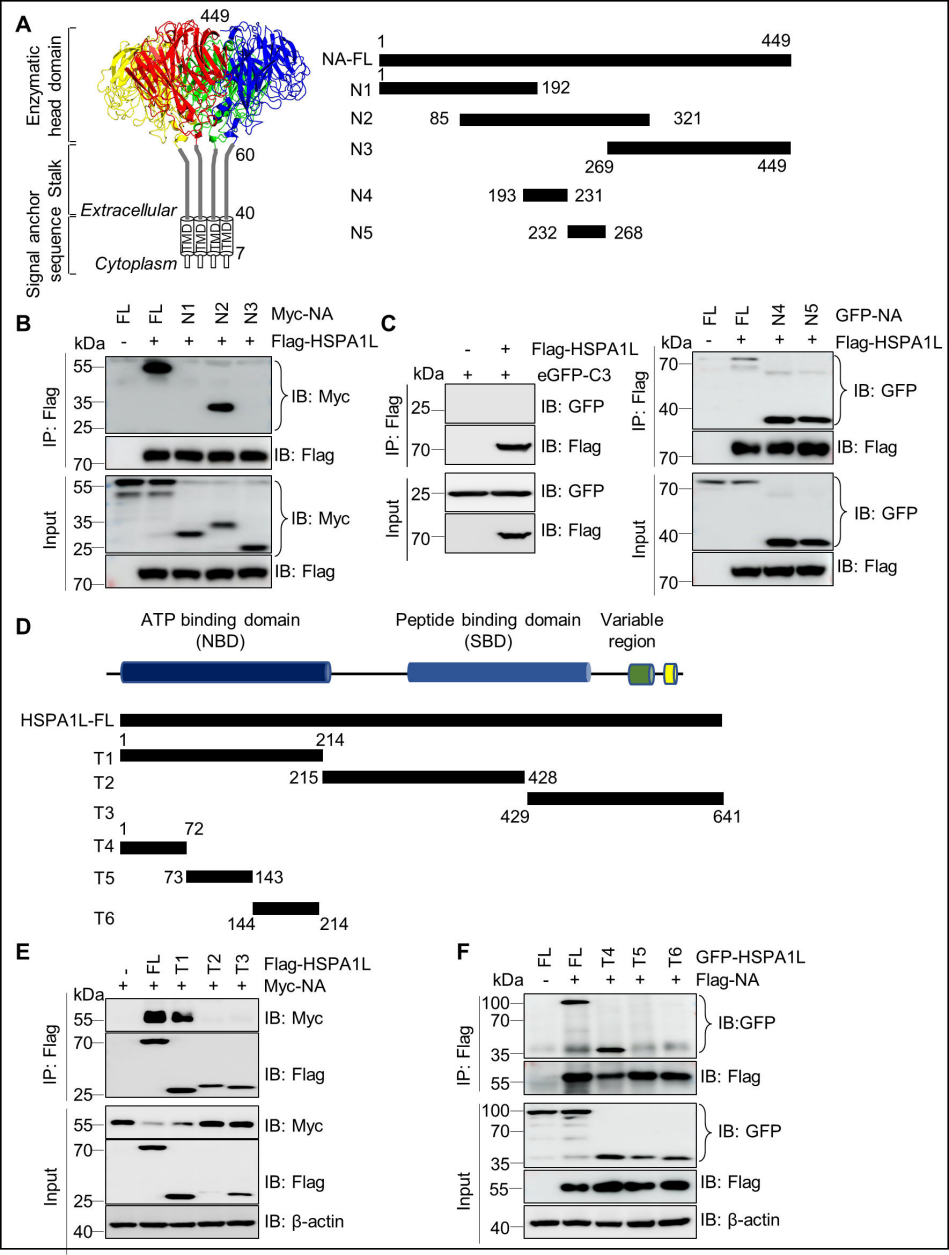
Domain requirements for HSPA1L-NA interaction. (**A**) The three domains of an NA tetramer are shown (Left). Schematic representation of NA or its truncated mutants (Right). (**B and C**) The residues 193–268 of NA are critical for binding to HSPA1L. HEK293T cells were co-transfected with Flag-HSPA1L and Myc-NA (**B**), GFP-tagged NA, its truncated mutants, or empty vector eGFP-C3 (**C**), immunoprecipitated with Flag beads and immunoblotted with indicated antibodies. (**D**) Schematic representation of HSPA1L or its truncated mutants. (**E and F**) The N-terminal residues of 1–72 of HSPA1L were required for interacting with NA. Flag-HSPA1L or its truncated mutants and Myc-NA (**E**), or Flag-NA and GFP-HSPA1L or its truncated mutants (**F**) were co-transfected into HEK293T cells, the cellular lysates were subjected to immunoprecipitation assays with Flag beads. The complexes were used for immunoblotting analysis using indicated antibodies.

NA protein consists of three domains: an enzymatic head domain, a stalk region, and an N-terminal transmembrane domain (TMD). TMD employs polar residues to autonomously assemble into an amphipathic tetramer, which stabilizes the stalk structure and facilitates the proper folding of the head domain (40, 41) (Fig. 2A). To determine the HSPA1L-interacting residues on NA, different H5N1-NA deletion mutants were generated and co-transfected with Flag-HSPA1L or corresponding empty vector into HEK293T cells. As shown in Fig. 2A through C, the amino acid region spanning residues 193–268 of NA is essential for binding HSPA1L. Similarly, through systematic truncation mutagenesis and co-IP assays, we further identified the N-terminal domain of HSPA1L (residues 1–72) as essential for its interaction with NA (Fig. 2D through F).

### HSPA1L restricts H5N1 replication

**Fig 3:**
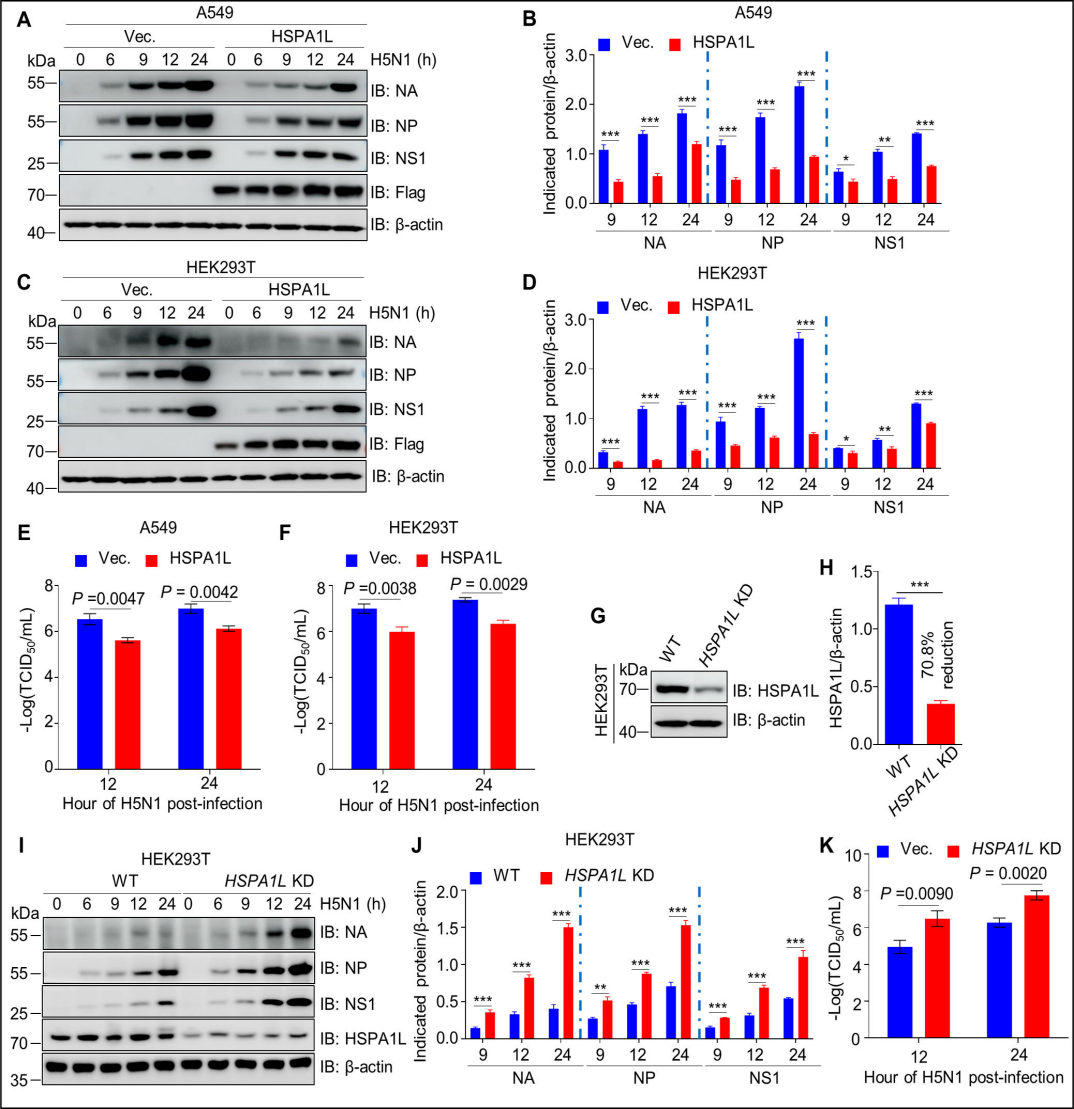
HSPA1L restricts H5N1 replication. (**A–F**) Overexpression of HSPA1L inhibits influenza A virus (IAV) H5N1 replication in A549 and HEK293T cells. A549 and HEK293T cells were separately transfected with an expression vector encoding HSPA1L or an empty vector control for 24 h. Subsequently, the cells were infected with the H5N1 virus (MOI = 3). At different time points post-infection, the cells were harvested and lysed in RIPA lysis buffer for western blotting analysis using mouse monoclonal antibodies (mAbs) against viral proteins NA, NP, and NS1 (**A and C**). Band intensities were quantified and normalized to β-actin (**B and D**). Viral titers in lysates from infected A549 (**E**) or HEK293T cells (**F**) were also determined by TCID_50_ assays. (**G and H**) Immunoblotting analysis of HSPA1L in WT and *HSPA1L* KD HEK293T cells (**G**), with quantification normalized to β-actin (**H**). (**I–K**) *HSPA1L* knockdown enhances H5N1 replication. *WT* and *HSPA1L* KD HEK293T cells were infected with the H5N1 virus (MOI = 3). At the indicated time points, the cells were harvested. The expression levels of viral proteins were analyzed by western blotting (**I**). The expression levels of NA, NP, and NS1 were quantified and normalized to β-actin (**J**). Viral titers in lysates from infected *HSPA1L* KD HEK293T cells (**K**) were assessed using the TCID_50_ assays.

To assess the role of HSPA1L in H5N1 replication, A549 and HEK293T cells were transfected with an HSPA1L expression plasmid or corresponding empty vector prior to H5N1 infection. Viral replication was monitored by detecting virus titer and the expression of viral proteins NA, NP, and NS1. As shown in Fig. 3A through F, the viral titer and expression of viral proteins were obviously inhibited in HSPA1L-overexpressing A549 and HEK293T cells compared with control cells. Subsequently, to further investigate the role of HSPA1L in H5N1 replication, the *HSPA1L* knockdown (*HSPA1L* KD) HEK293T cells were generated by using the Cas9 enzyme (Fig. 3G and H). Afterward, the replication efficiency of H5N1 was determined in wild-type (WT) and *HSPA1L* KD cells. As shown in Fig. 3I and K, viral proteins and virus titers were enhanced in *HSPA1L* KD cells compared with WT cells. Altogether, these findings indicated that the host protein HSPA1L expression contributes to control H5N1 infection.

### HSPA1L promotes the autophagic degradation of NA

**Fig 4:**
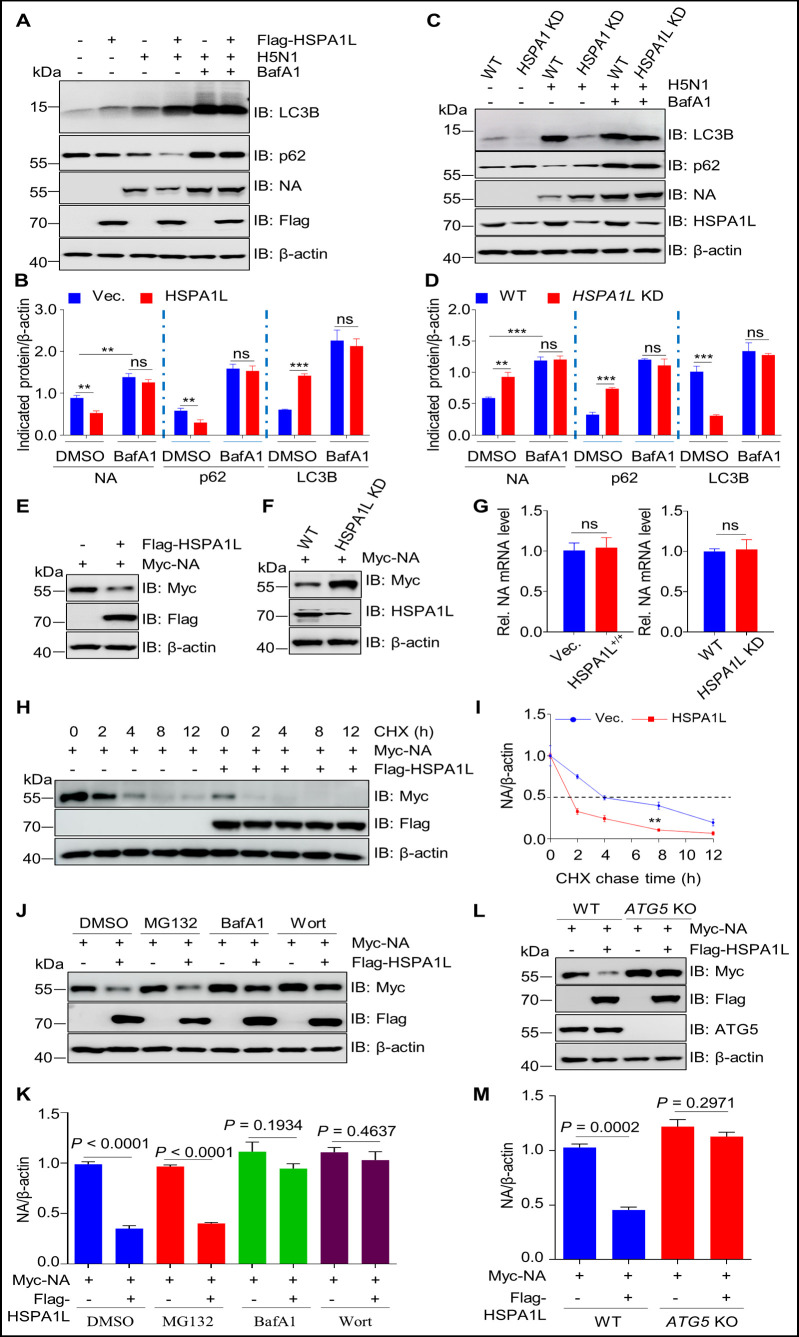
HSPA1L mediates the autophagic degradation of NA. (**A and B**) HSPA1L overexpression accelerates IAV-induced autophagy. A549 cells were transfected with Flag-HSPA1L or corresponding empty vector. At 18 h post-transfection, the cells were treated with BafA1 (200 nM) or DMSO (control) for 6 h, followed by infection with H5N1 (MOI = 3). At 18 h after infection, the cells were harvested and subjected to immunoblotting assays using indicated antibodies (**A**). Band intensities from three independent biological replicates in (**A**) were quantified and normalized to β-actin (**B**). (**C and D**) *HSPA1L* knockdown inhibits autophagy induced by IAV infection. WT and *HSPA1L* KD HEK293T cells were treated with BafA1 (200 nM) or DMSO for 6 h. Subsequently, the cells were infected with H5N1 (MOI = 3) for 18 h. The cellular lysates were then subjected to immunoblotting using indicated antibodies (**C**). Band intensities from three independent biological replicates in (**C**) were quantified and normalized to β-actin (**D**). (**E–G**) HSPA1L inhibits the stability of NA. HEK293T cells were co-transfected with Myc-NA and Flag-HSPA1L or an empty vector control (**E**). Separately, Myc-NA was transfected into WT and *HSPA1L* KD HEK293T cells (**F**). At 24 h post-transfection, the cells were harvested, and the expression levels of the NA protein and mRNA were assessed by Western blot and qPCR assays (**G**). (**H and I**) HSPA1L overexpression shortens the half-life of NA. Vectors expressing Myc-NA and Flag-HSPA1L or the corresponding empty vector were co-transfected into HEK293T cells for 24 h. The resultant cells were treated with cycloheximide (CHX, 100 µg/mL). At indicated time points, the cells were harvested and subjected to immunoblotting analysis (**H**). The intensities of the bands on the immunoblots from three independent experiments were quantified and normalized with β-actin (**I**). Statistical significance was analyzed by two-way ANOVA. (**J and K**) HSPA1L facilitates NA autophagic degradation. Vectors expressing Myc-NA and Flag-HSPA1L or the corresponding empty vector were co-transfected into HEK293T cells for 24 h, followed by treatment for 6 h with BafA1 (200 nM) or Wort (20 µM), and MG132 (10 µM). The cellular lysates were used for immunoblotting analysis (**J**). The intensities of the bands on the immunoblots from three independent experiments were quantified and normalized with β-actin (**K**). (**L and M**) Immunoblotting analysis of Myc-NA in cellular lysates from WT and *ATG5* knockout cells transfected with vectors expressing Flag-HSPA1L. WT and *ATG5* knockout HEK293T cells were co-transfected with vectors expressing Myc-NA and Flag-HSPA1L or corresponding empty vector for 24 h. The cellular lysates were used for immunoblotting analysis (**L**). The intensities of the bands on the immunoblots from three independent experiments were quantified and normalized with β-actin (**M**).

Accumulating evidence indicates that HSP family members, including Hsp70 and Hsp90, regulate autophagic flux through distinct mechanisms (42, 43). Our data demonstrate that HSPA1L overexpression enhances autophagic flux in H5N1-infected A549 cells, as evidenced by increased LC3-II accumulation and concomitant downregulation of p62 protein levels compared to vector controls (Fig. 4A and B). Notably, these effects were abolished upon treatment with the autophagy inhibitor bafilomycin A1 (BafA1) (Fig. 4A and B). Conversely, *HSPA1L* knockdown significantly decreased LC3-II levels and increased p62 accumulation, consistent with impaired autophagosome clearance (Fig. 4C and D). Taken together, these findings indicate that HSPA1L promotes the autophagic flux induced by H5N1 infection. The enhancement effect of HSPA1L on H5N1-induced autophagic flux led us to investigate whether the expression of NA was regulated by HSPA1L. To investigate HSPA1L’s regulatory effects on NA, we first detected NA protein expression by Western blot and analyzed NA mRNA levels via quantitative PCR (qPCR). As demonstrated in Fig. 4E and F, HSPA1L overexpression reduced H5N1-NA protein abundance, while *HSPA1L* knockdown conversely increased NA levels. Notably, qPCR analysis revealed no significant changes in *NA* mRNA (Fig. 4G), which suggested that HSPA1L is involved in the post-transcriptional regulation of NA. Therefore, we employed the protein synthesis inhibitor cycloheximide (CHX) to investigate the impacts of HSPA1L on the stability of NA. The results demonstrated that the half-life of H5N1-NA was significantly shortened in HSPA1L-overexpressing cells compared with that of the control (Fig. 4H and I), indicating that HSPA1L contributes to accelerate the degradation of NA.

Autophagy and the ubiquitin-proteasome system are two major intracellular degradative pathways (44). To examine if HSPA1L facilitated NA degradation by autophagy, an experiment using autophagy inhibitors, bafilomycin A1 (BafA1) and wortmannin (Wort), was conducted. As demonstrated in Fig. 4J and K, HSPA1L-mediated NA degradation was almost blocked by the autophagy inhibitors Wort and BafA1 but not by the proteasome inhibitor MG132. We further assessed the role of HSPA1L in *ATG5* KO cells, in which the autophagy is impaired. We found that *ATG5* knockout almost completely blocked NA degradation induced by HSPA1L (Fig. 4L and M). Collectively, these findings demonstrate that HSPA1L promotes autophagosome-dependent degradation of the H5N1-NA protein.

### The selective autophagy receptor NBR1 mediates HSPA1L-driven degradation of H5N1-NA

**Fig 5:**
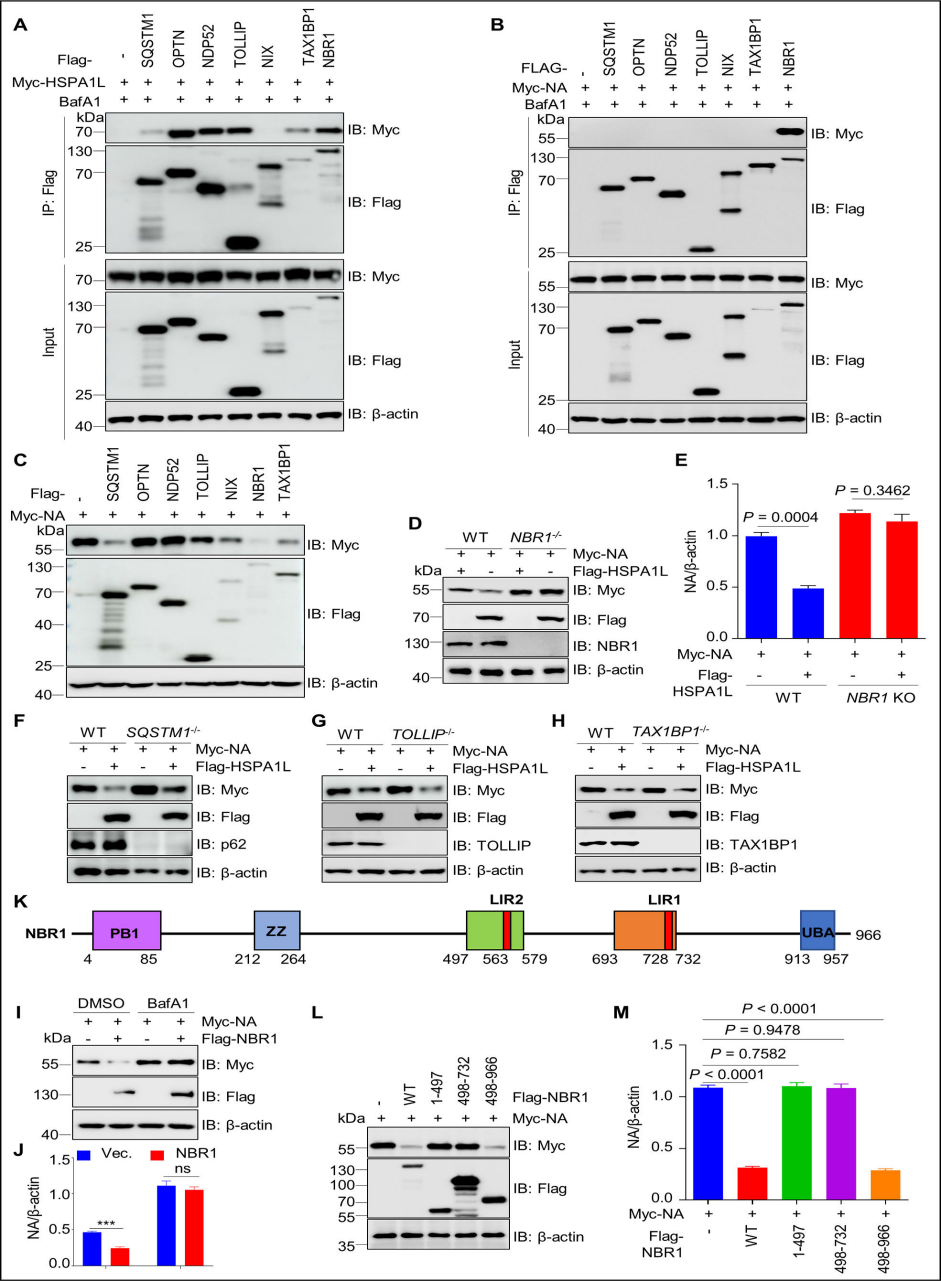
NBR1 is required for HSPA1L-mediated degradation of NA. (**A**) HSPA1L interacts with SQSTM1, OPTN, NDP52, TOLLIP, NBR1, and TAX1BP1, but not with NIX. Vectors expressing Myc-HSPA1L and different Flag-tagged autophagy receptors were co-transfected into HEK293T cells for 36 h, respectively. The cellular lysates were subjected to immunoprecipitation assays with Flag beads, followed by an immunoblotting assay using the indicated antibodies. (**B**) NA interacts strongly with NBR1, but not with SQSTM1, OPTN, NDP52, NIX, TOLLIP, and TAX1BP1. Vectors expressing Myc-NA and different Flag-tagged autophagy receptors were co-transfected into HEK293T cells for 36 h, respectively. Cellular lysates were subjected to an immunoprecipitation assay using Flag beads, followed by an immunoblotting assay using the indicated antibodies. (**C**) SQSTM1, TOLLIP, NBR1, or TAX1BP1 overexpression promotes degradation of NA. Myc-NA and different Flag-tagged autophagy receptors or corresponding empty vector were co-transfected into HEK293T cells for 24 h. The cellular lysates were subjected to western blotting assays using indicated antibodies. (**D–H**) *NBR1*, but not *SQSTM1*, *TOLLIP*, and *TAX1BP1*, knockout almost abolished HSPA1L-mediated the degradation of NA. Myc-NA and Flag-HSPA1L or corresponding empty vector were co-transfected into WT, *NBR1*^−/−^ (**D and E**), *SQSTM1*^−/−^ (**F**), *TOLLIP*^−/−^ (**G**), or *TAX1BP1*^−/−^ (**H**) HEK293T cells for 24 h. The cellular lysates were used for immunoblotting analysis with indicated antibodies. The intensities of the bands on the immunoblots from three independent experiments in (**D**) were quantified and normalized to β-actin (**E**). (**I**) NBR1 promotes NA autophagic degradation. Myc-NA and Flag-NBR1 or the corresponding empty vector were co-transfected into HEK293T cells for 24 h, followed by treatment for 6 h with BafA1 (200 nM). The lysates were used for immunoblotting analysis. (**J**) The intensities of the bands on the immunoblots from three independent experiments in (**I**) were quantified and normalized to β-actin. (**K**) Schematic representation of NBR1. (**L**) Vectors expressing Flag-NBR1 and its different deletion mutants along with Myc-NA were separately co-transfected into HEK293T cells for 24 h. The cells were harvested and subjected to immunoblotting assay using the indicated antibodies. (**M**) The intensities of the bands on the immunoblots from three independent experiments in (**L**) were quantified and normalized to β-actin.

A growing evidence suggests that autophagy receptors play important roles in delivering substrates to the autophagosome for degradation (44–47). To determine whether HSPA1L-mediated degradation of NA is associated with cargo receptors, we investigated the interaction between HSPA1L and multiple selective autophagic receptors, including SQSTM1/p62, optineurin (OPTN), calcium-binding and coiled-coil domain 2 (CALCOCO2/NDP52), Toll-interacting protein (TOLLIP), NIP3-like protein X (NIX), BRCA1 gene 1 (NBR1), and Tax1 binding protein 1 (TAX1BP1). As shown in Fig. 5A, HSPA1L interacted with multiple cargo receptors but not with NIX. Notably, H5N1-NA interacted only with NBR1 and not with SQSTM1, OPTN, NDP52, TOLLIP, NIX, or TAX1BP1 (Fig. 5B). Moreover, NA expression levels were lower in NBR1-overexpressing cells compared to cells expressing other autophagy receptors (e.g., SQSTM1, OPTN, NDP52) (Fig. 5C). These results suggest that HSPA1L-mediated autophagic degradation of NA might be dependent on NBR1. Subsequently, to address this, NBR1, p62, TOLLIP, and TAX1BP1 knockout cells were separately generated. Western blot assays showed that HSPA1L-induced NA degradation was significantly blocked in the absence of NBR1 but not p62, TOLLIP, and TAX1BP1 (Fig. 5D through H), indicating that NBR1 is essential for HSPA1L-mediated NA degradation. Furthermore, NBR1-mediated NA degradation was almost completely blocked by the autophagy inhibitor BafA1 (Fig. 5I and J), demonstrating that NBR1 promotes autophagic degradation of NA. NBR1 exerts its degradative biological effects mainly through two critical domains: the LIR domain associates with LC3, and the UBA domain recognizes ubiquitinated substrates (48). To determine which domain is required for NA degradation, we performed deletion mutagenesis and found that NBR1 lacking both the LIR and UBA domains failed to promote NA degradation (Fig. 5K through M), suggesting NBR1 may mediate autophagic degradation of ubiquitinated NA.

### HSPA1L ubiquitinates the residue K242 within H5N1-NA

**Fig 6:**
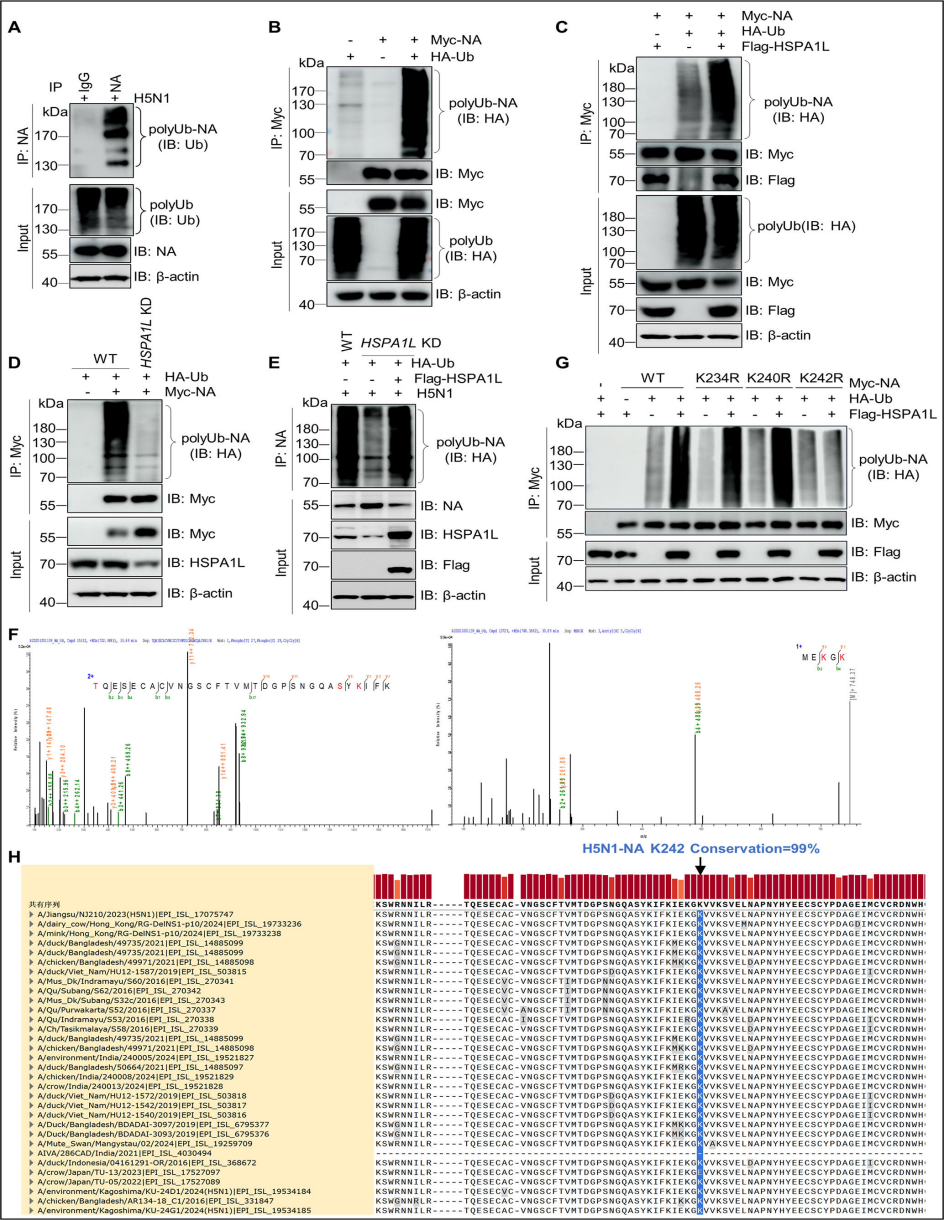
HSPA1L ubiquitinates the residue K242 of NA. (**A**) Viral protein NA can be ubiquitinated during H5N1 infection. H5N1-infected A549 cells at a MOI of 3. At 18 h after infection, cellular lysates were used for NA immunoprecipitation and western blotting with anti-ubiquitin and anti-NA mouse mAbs. (**B**) NA is ubiquitinated during transfection. HEK293T cells were co-transfected with vectors expressing Myc-NA and HA-Ub for 48 h. Cellular lysates were subjected to an immunoprecipitation assay with Myc beads and western blotting with the indicated antibodies. (**C**) HSPA1L overexpression promotes the ubiquitination of NA. The lysates from HEK293T cells overexpressing Myc-NA and Flag-TRAF6 along with HA-Ub were immunoprecipitated with Myc beads and immunoblotted using the indicated antibodies. (**D**) *HSPA1L* knockdown inhibits ubiquitination of NA. Myc-NA and HA-Ub were separately co-transfected into WT and *HSPA1L* KD HEK293T cells for 36 h, and then the cellular lysates were immunoprecipitated with Myc beads and immunoblotted using the indicated antibodies. (**E**) The restoration of HSPA1L blocks the inhibition of *HSPA1L* knockdown on NA ubiquitination. WT and *HSPA1L* KD HEK293T cells transfected with Flag-HSPA1L or corresponding empty vector were transfected with HA-Ub for 24 h, followed by H5N1 infection for 18 h. The cellular lysates were immunoprecipitated with Myc beads and immunoblotted using the indicated antibodies. (**F**) Identification of ubiquitination sites on NA by mass spectrometry analysis. A549 cells were infected with H5N1 (MOI = 3) for 18 h. The cellular lysates were immunoprecipitated using mouse anti-NA monoclonal antibody (mAb). The immunoprecipitated complexes were subjected to mass spectrometry analysis. The MS spectra of the selected ubiquitinated NA peptides, TQESECACVNGSCFTVMTDGPSNGQASYK^234^IFK and MEK^240^GK^242^, are shown. (**G**) HSPA1L promotes the ubiquitination of NA at K242. HEK293T cells were co-transfected with vectors expressing Myc-NA or its mutants, HA-Ub, and Flag-HSPA1L for 36 h. Then, cellular lysates were immunoprecipitated and subjected to immunoblotting assays using indicated antibodies. (**H**) Conservation analysis of the K242 residue in H5N1 NA. A total of 2,528 H5N1 NA sequences from Asia (2015–2025) were retrieved from GISAID. Multiple sequence alignment was performed using MAFFT (v7.490), and results were visualized in SnapGene (v7.2.1).

Given that ubiquitin chains on substrates act as a critical recognition signal for selective autophagy receptors, we speculated that NA is a ubiquitinated protein. To identify our hypothesis, we first detected if NA undergoes ubiquitination during viral infection. As shown in Fig. 6A, H5N1-NA could be ubiquitylated during viral infection. Moreover, exogenous NA also could undergo ubiquitination (Fig. 6B). Given that HSPA1L mediates NBR1-dependent autophagic degradation of NA (Fig. 5D and E), we investigated its role in regulating NA ubiquitination. Strikingly, HSPA1L overexpression markedly enhanced NA ubiquitination (Fig. 6C), whereas *HSPA1L* knockdown significantly reduced ubiquitinated NA levels (Fig. 6D). Furthermore, re-expression of HSPA1L fully eliminated the suppression of NA ubiquitination caused by *HSPA1L* knockdown (Fig. 6E). Taken together, our findings provide evidence that HSPA1L enhances NA ubiquitination. Subsequently, to identify HSPA1L-regulated ubiquitination sites on NA, we first mapped ubiquitinated residues within NA using mass spectrometry. Three lysine (K) residues (K234, K240, and K242) were identified as ubiquitination sites in NA (Fig. 6F). We next generated NA mutants with single K-to-arginine (R) substitutions (K234R, K240R, K242R) and assessed the impact of HSPA1L overexpression on their ubiquitination. Results showed that HSPA1L overexpression markedly enhanced ubiquitination of WT, K234R, and K240R mutant NA but had no significant effect on mutant K242R ubiquitination (Fig. 6G), indicating that HSPA1L mediated the ubiquitination of the residue K242 of NA. Strikingly, K242 in H5N1-NA exhibited 99% conservation across strains (Fig. 6H), suggesting that HSPA1L may exhibit broad-spectrum antiviral activity against diverse H5N1 strains.

### HSPA1L ubiquitinates the residue K242 of NA to inhibit H5N1 replication

**Fig 7:**
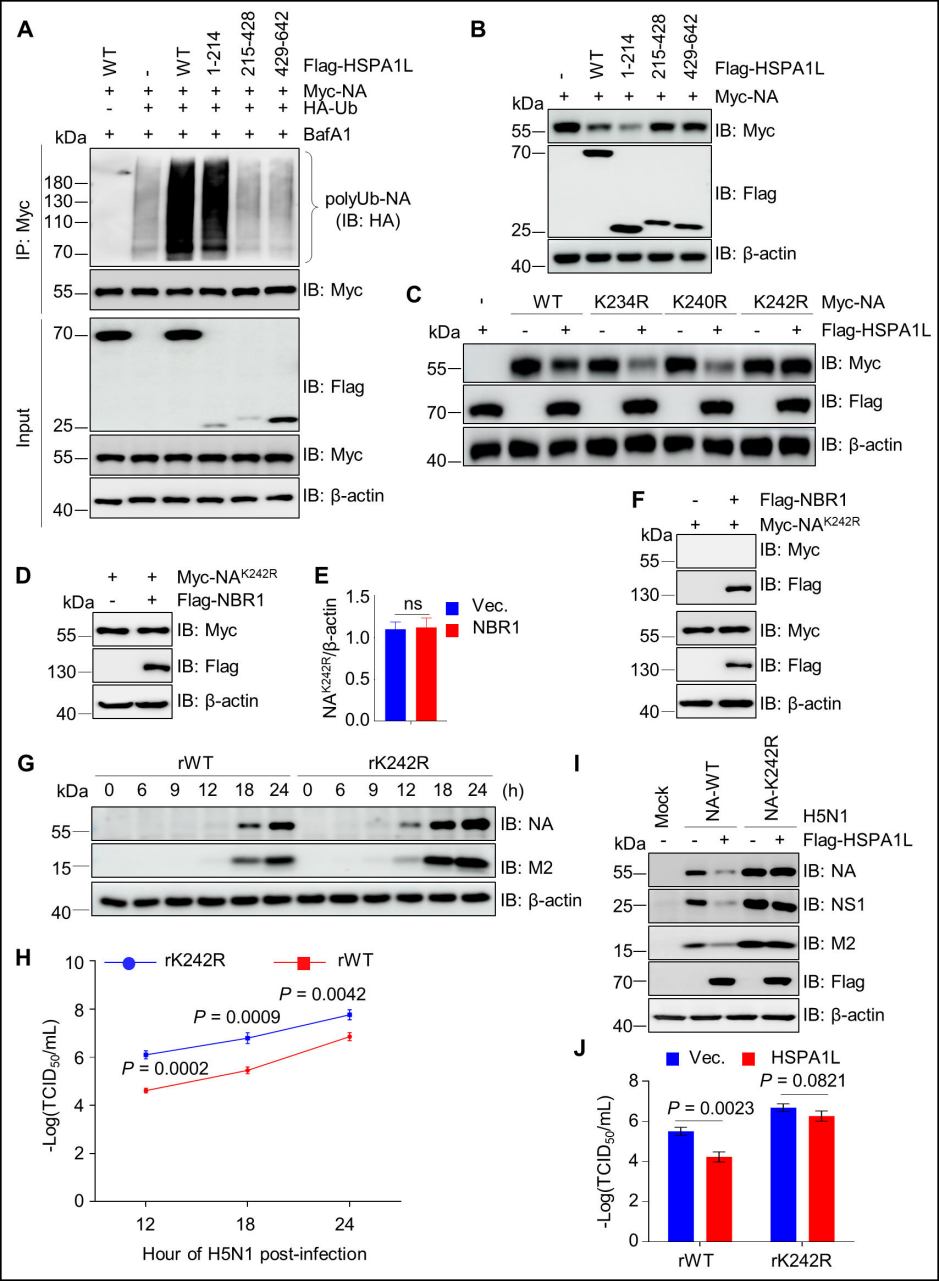
HSPA1L restricts H5N1 replication by promoting the ubiquitination of NA at K242. (**A**) The N-terminal residues of 1–214 of HSPA1L are necessary for mediating NA ubiquitination. Myc-NA, HA-Ub, and Flag-HSPA1L or its truncated mutants were co-transfected into HEK293T cells for 30 h, followed by treatment with BafA1 (200 nM) for 6 h. The cellular lysates were immunoprecipitated with Myc beads and immunoblotted using indicated antibodies. (**B**) The N-terminal residues of 1–214 were required for HSPA1L to degrade NA. Myc-NA and Flag-HSPA1L or its truncated mutants were co-transfected into HEK293T cells for 24 h. The cellular lysates were subjected to immunoblotting analysis. (**C**) The ubiquitination site K242 of NA is crucial for HSPA1L to degrade NA. HEK293T cells were co-transfected with Myc-NA or its mutants and Flag-HSPA1L or corresponding empty vector for 24 h. The cellular lysates were subjected to western blotting assays using indicated antibodies. (**D and E**) NBR1 overexpression does not affect the protein levels of the NA^K242R^ mutant. HEK293T cells were co-transfected with Myc-NA^K242R^ and Flag-NBR1 or an empty vector control for 24 h. The lysates were subjected to immunoblotting analysis with indicated antibodies (**D**). The intensities of the bands on the immunoblots from three independent experiments in (**D**) were quantified and normalized to β-actin (**E**). (**F**) NBR1 does not interact with NA^K242R^ mutant. Flag-NBR1 or corresponding empty vector and Myc-NA^K242R^ mutant were co-transfected into HEK293T cells for 36 h. The cellular lysates were immunoprecipitated with Flag beads and immunoblotted using indicated antibodies. (**G**) A549 cells were infected with recombinant viruses rWT and rK242R (MOI = 1), respectively. At different time points, the cells were harvested and subjected to Western blot with anti-NA, anti-NS1, and anti-M2 antibodies. β-actin was used as the loading control. (**H**) A549 cells were infected with recombinant viruses rWT and rK242R (MOI = 1), respectively. At the indicated time after infection, the cells were harvested and subjected to virus titer detection by the TCID_50_. (**I and J**) HSPA1L inhibits H5N1 replication through modulating the ubiquitination of NA at K242. A549 cells were transfected with Flag-HSPA1L or corresponding empty vector for 24 h, followed by infection with recombinant viruses rWT and rK242R (MOI = 3) for 12 h, respectively. The cells were harvested and subjected to Western blot analysis (**I**) and viral titer detection by the TCID_50_ (**J**).

To determine the HSPA1L domain responsible for mediating NA ubiquitination, we analyzed the effects of HSPA1L deletion mutants on NA ubiquitination. Results showed the N-terminal region of HSPA1L (residues 1–214), which is required for NA binding, was essential to promote NA ubiquitination (Fig. 7A). Furthermore, this domain was necessary for HSPA1L to degrade NA (Fig. 7B). To identify whether HSPA1L-mediated NA degradation depends on its regulation of NA ubiquitination, we analyzed the effects of HSPA1L overexpression on the expression of different NA mutants by Western blot. As shown in Fig. 7C, HSPA1L overexpression significantly reduced the levels of WT, K234R, and K240R mutant NA but had no effect on the K242R mutant, demonstrating that ubiquitination of the residue K242 is essential for NA degradation mediated by HSPA1L. Additionally, given that HSPA1L-mediated degradation of NA is NBR1-dependent (Fig. 5D and E), we further investigated whether NBR1 promotes NA degradation by recognizing its ubiquitin chains at the K242 site. The results demonstrated that NBR1 failed to degrade the ubiquitination-deficient mutant NA^K242R^ and showed no binding interaction with it (Fig. 7D through F), suggesting that NBR1 specifically recognizes ubiquitinated NA at K242 to mediate its autophagic degradation. Subsequently, to determine whether the K242 residue of NA is required for HSPA1L-mediated suppression of H5N1 replication, we rescued recombinant viruses H5N1 carrying wild-type (rWT) or the K242R mutant NA (rK242R). A549 cells infected with rWT or rK242R viruses showed significantly higher levels of viral protein expression (Fig. 7G) and virus titers (Fig. 7H) for rK242R compared to rWT, indicating that the residue K242 on NA is critical for restricting H5N1 replication. Furthermore, in HSPA1L-overexpressing A549 cells, HSPA1L potently suppressed rWT replication but failed to inhibit rK242R virus (Fig. 7I and J), indicating that HSPA1L-mediated antiviral activity strictly depends on NA ubiquitination at K242.

### HSPA1L acts as a broad-spectrum restrictor of influenza A virus

**Fig 8:**
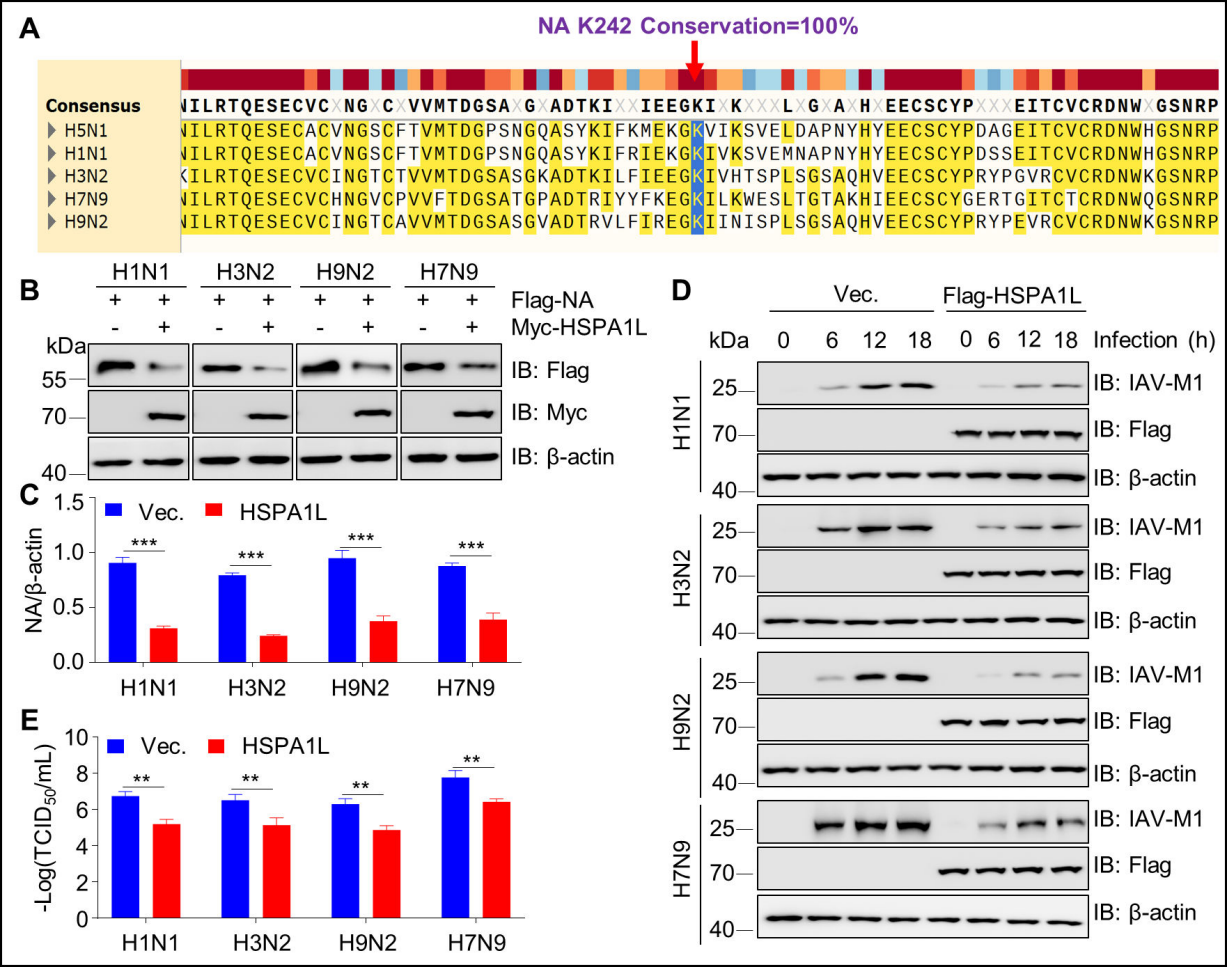
HSPA1L inhibits replication of multiple influenza A virus subtypes. (**A**) Conservation of the K242 residue in NA across influenza A virus subtypes. NA sequences from H1N1 (EF190976.1), H5N1 (GQ227369.1), H3N2 (GU086091.1), H9N2 (KF042145.1), and H7N9 (KC853765.1) were retrieved from NCBI. Sequences were aligned using MAFFT (v7.490) and visualized in SnapGene (v7.2.1). (**B**) HSPA1L overexpression reduces NA expression in multiple influenza A virus subtypes. HEK293T cells were co-transfected with Flag-tagged NA (H1N1, H3N2, H9N2, or H7N9) and Myc-HSPA1L or corresponding empty vector for 24 h. Cell lysates were analyzed by immunoblotting with the indicated antibodies. (**C**) Quantification of NA expression levels. Band intensities from three independent experiments in (**B**) were quantified and normalized to β-actin. (**D and E**) HSPA1L overexpression suppresses influenza A virus replication. HEK293T cells transfected with HSPA1L or an empty vector for 24 h were infected with H1N1, H3N2, H9N2, or H7N9 (MOI = 3). At indicated time points, cells were lysed for immunoblotting with mouse anti-IAV-M1 antibody (**D**). The cellular lysates from IAV-infected for 18 h were used for viral titer measurement (**E**).

To determine whether HSPA1L regulates other influenza A virus subtypes (e.g., H1N1, H3N2, H9N2, and H7N9) similar to its role in H5N1, we aligned the NA proteins of these subtypes. The results revealed that the K242 site is 100% conserved across all tested subtypes (Fig. 8A), suggesting that HSPA1L may modulate multiple influenza A virus subtypes through a common mechanism. Consistent with this hypothesis, HSPA1L overexpression significantly reduced NA protein levels in H1N1, H3N2, H9N2, and H7N9 viruses (Fig. 8B and C). Moreover, HSPA1L overexpression markedly decreased both viral protein M1 expression and viral titers (Fig. 8D and E). Collectively, these findings demonstrate that HSPA1L broadly inhibits influenza A virus replication across diverse subtypes.

## DISCUSSION

**Fig 9:**
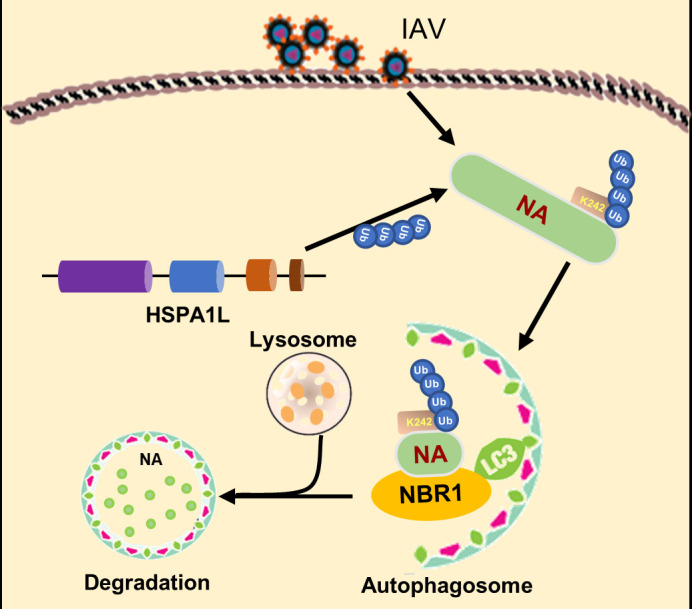
Schematic depiction of the proposed mechanism by which HSPA1L counteracts IAV infection. HSPA1L interacts with IAV NA protein and promotes its ubiquitination at K242, leading to its NBR1-dependent autophagic degradation and subsequent suppression of IAV replication.

Host cells have developed a diverse array of defense mechanisms to counteract influenza A virus infection. Among these, the most well-characterized antiviral systems are the broadly acting pattern-recognition receptors, such as Toll-like receptors (TLRs) and RIG-I-like receptors (RLRs) (49–51). These receptors play a critical role in detecting microbial nucleic acids, initiating the host’s immune response. These innate antiviral defenses indirectly inhibit infection by triggering signaling cascades that lead to the production of interferons and other antiviral effector molecules. In contrast, as demonstrated in this study, HSPA1L functions in a more targeted manner by directly detecting an IAV “danger” signal and limiting infection in a highly specific way. This study uncovers a previously unrecognized antiviral mechanism in which the host HSPA1L restricts IAV replication by targeting the viral protein NA for ubiquitination at K242 and subsequent NBR1-dependent autophagic degradation (Fig. 9). Our findings establish HSPA1L as a critical component of intrinsic defense against IAV, operating through direct engagement with NA and site-specific ubiquitination of the viral protein.

HSP70 family members are increasingly recognized for their dual roles in viral infections, either enhancing or inhibiting viral replication depending on the pathogen and cellular context (52, 53). While prior studies implicated HSP70 in modulating viral replication or immune signaling, HSPA1L’s role in antiviral defense remained unexplored. Here, we demonstrate that HSPA1L interacts with and promotes ubiquitination of H5N1-NA at K242, marking it for autophagic degradation via the cargo receptor NBR1, thereby inhibiting viral replication. This mechanism parallels recent findings showing that HSP70, another HSP70 family member, binds to the hexon protein of Fowl Adenovirus Serotype 4 and facilitates its autophagic degradation to suppress viral replication (31). Notably, our study provides the first mechanistic insight into how HSP70 family proteins exploit the host ubiquitination system to trigger autophagic degradation as an antiviral strategy. Moreover, we identify HSPA1L as a novel promising target for developing antiviral therapies against IAV. However, the precise mechanism by which HSPA1L promotes NA ubiquitination remains unclear. Specifically, it is unknown whether HSPA1L recruits an E3 ubiquitin ligase to ubiquitinate NA in a manner similar to HSP70, which recruits carboxyl terminus of Hsc-70-interacting protein (CHIP) to mediate p63 ubiquitination and degradation (20), or whether it possesses intrinsic E3 ligase activity. This question warrants further investigation.

The host ubiquitin (Ub) system serves as a critical antiviral defense mechanism, primarily through two major degradation pathways: the ubiquitin-proteasome system (UPS) and autophagy (48, 54–56). While substrates modified with K48-linked polyubiquitin chains are primarily degraded via the ubiquitin-proteasome pathway (55), those conjugated with K48-, K63-, K27-, K33-, or K11-linked polyubiquitin chains can also be recognized by selective autophagy receptors (44, 48, 54, 57–59), leading to autophagosome-dependent clearance. Although previous studies have established that TRIM family proteins (TRIM21/22/32/35) restrict IAV by targeting viral proteins (M1, NP, PB1, PB2) for proteasomal degradation (55, 56, 60, 61), our study identifies an alternative antiviral strategy. We demonstrate that HSPA1L, unlike canonical TRIM-mediated proteasomal targeting, specifically promotes ubiquitination of viral protein NA to trigger NBR1-dependent autophagic degradation. This mechanism parallels recent findings showing NBR1-mediated autophagic clearance of K27-ubiquitinated MAVS and K48-ubiquitinated TBK1 (48, 54). However, whether HSPA1L promotes K27- or K48-linked polyubiquitination of NA to facilitate NBR1 recognition and subsequent autophagic degradation remains to be further investigated in the future.

The identification of H5N1 NA K242 as the key ubiquitination site provides mechanistic clarity to this antiviral activity. The resistance of the K242R mutant virus H5N1 to HSPA1L-mediated suppression confirms that ubiquitination at this residue is indispensable for restricting NA stability and viral replication. Notably, K242 resides in a conserved region of H5N1-NA and other IAV subtypes, suggesting that HSPA1L may exert broad-spectrum activity against multiple IAV subtypes. It is well established that host-adaptive mutations are critical for viral fitness and cross-species transmission. IAV can employ multilayered adaptive mechanisms to evade host antiviral defenses (55, 62–68). This raises an important question: could IAV evolve compensatory mutations under selective pressure from HSPA1L to escape host defenses and achieve multi-host infection? This intriguing possibility warrants further investigation in future studies.

In conclusion, we delineate a unique antiviral axis wherein HSPA1L exploits the host ubiquitination and autophagy machinery to degrade IAV NA, unveiling a potential target for therapeutic intervention against IAV. These findings expand the functional repertoire of heat shock proteins in antiviral defense and underscore the importance of post-translational modifications in host-pathogen conflicts.

## MATERIALS AND METHODS

### Cells and virus

Human embryonic kidneys (HEK) 293T (CRL-11268, ATCC, Manassas, VA, USA) and human lung epithelial A549 (CRM-CCL-185, ATCC) cells were routinely cultured in Dulbecco’s modified Eagle’s medium (DMEM, Gibco, Carlsbad, CA) containing 10% fetal bovine serum (FBS). *TOLLIP* knockout HEK293T cells were generously provided by Dr. Jianwei Zhou (Yangzhou University). *ATG5* and *SQSTM1/p62* knockout HEK293T cells were maintained in our laboratory and have been described previously (44). A/Puerto Rico/8/34(PR8), A/swine/Guangdong/(H3N2), A/Chicken/Jiande/09/2009 (H9N2), and A/Hangzhou/1/2013(H7N9) were stored in our laboratory (55). A/Duck/Fujian/2018 (H5N1) was reserved in Fujian Academy of Agricultural Sciences Institute of Animal Husbandry and Veterinary Medicine, China.

### Antibodies and reagents

Rabbit anti-HSPA1L (A1856), anti-p62 (A19700), TAX1BP1 (A19587), and anti-NBR1 (A3949) were offered by ABclonal (Wuhan, China). Mouse anti-TOLLIP (68170-1-Ig) was purchased by Proteintech. Rabbit anti-LC3B (2775) and mouse anti-Ubiquitin (3936) were supplied by Cell Signaling Technology. Rabbit anti-ATG5 (ET1611-38), anti-His Tag (0812), anti-Myc (R1208-1), anti-Flag (0912-1) and anti-GFP (ET1607-31), and mouse anti-GST Tag (EM80701) and anti-β-actin (M1210-2) were purchased from HuaAn Biotech (Hangzhou, China). Mouse anti-Flag (F1804) was obtained from Sigma-Aldrich (St. Louis, MO, USA). Mouse monoclonal antibodies (mAbs) against influenza viral proteins were prepared and stored by our laboratory. Horseradish peroxidase (HRP)-labeled goat anti-mouse (074-1806) or anti-rabbit (074-1506) IgG was purchased from Kirkegaard & Perry Laboratories (Milford, MA, USA). Protein A/G PLUS-Agarose (sc-2003) was offered by Santa Cruz Biotechnology. Flag beads (FNM-250-10K) and MYC beads (MNM-125-5000) were supplied by LABLEAD (Beijing, China). Ni-NTA Agarose (30230) was purchased from QIAGEN (Germany). Glutathione (GST) Resin (L00206) was supplied by GenScript (New Jersey, USA). Reduced glutathione (A100399-0005) was purchased from Sangon Biotech (Shanghai, China). NP-40 lysis buffer (P0013F) was purchased from Beyotime (Shanghai, China). Phenylmethylsulfonyl fluoride (PMSF) (P8340) was supplied by Solarbio (Shanghai; China). Bafilomycin A1(BafA1; HY-100558), Wortmannin (Wort, HY-10197), MG-132 (HY-13259), and Cycloheximide (CHX, HY-12320) were purchased from MedChemExpress (Monmouth Junction, NJ, USA).

### Plasmids and transfection

The DNA fragment encoding full-length and truncated variants HSPA1L and NA were separately amplified from HEK293T cells and A/Duck/Fujian/2018 (H5N1), A/Puerto Rico/8/34(PR8), A/swine/Guangdong/ (H3N2), A/Chicken/Jiande/09/2009 (H9N2), and A/Hangzhou/1/2013(H7N9) strain, and were separately subcloned into vectors pCMV-Myc-N (635689; Clontech, Palo Alto, CA, USA), pCMV-Flag-N (635688; Clontech), pET-28a-C (69864-3; Novagen, Madison, WI, USA), pEGFP-C3 (6082-1, Clontech), and pGEX-4T-1 (27-4580-01; GE Healthcare Biosciences, Piscataway, NJ, USA). The resultant plasmids were Flag-HSPA1L, Myc-HSPA1L, pET-28a-HSPA1L, Flag-H1/H3/H5/H7/H9-NA, Myc-NA, GFP-HSPA1L, and pGEX-4T-1-NA. Rescue plasmids of H5N1 virus were stored in our laboratory and have been described previously (55). Mutations of Myc-NA and the vRNA-mRNA bidirectional transcription vector pLLBA/G-H5N1-NA were created by a site-specific mutation method using the WT plasmid as the template. HEK293T and A549 cells were separately transfected using Polyethylenimine HCl MAX, Linear (P4000; LABLEAD, Beijing, China) and JetPRIME (PT-114-15; New York, NY, USA) transfection reagent according to the manufacturers’ instructions. The primers used for cloning and mutations are listed in Table 1.

**TABLE 1:** Primers used for cloning and mutation construction

Name	Forward sequence (5′−3′)	Reverse sequence (5′−3′)
*HSPA1L-*FL	ATGGCTACTGCCAAGGGAATCG	TTAATCTACTTCTTCAATTG
*HSPA1L*-T1	ATGGCTACTGCCAAGGGAATCG	TTATATGGTCAGAATTGACAC
*HSPA1L*-T2	ATGGATGATGGGATTTTTGAG	TTACTGTGTCTGCTTGGTGGG
*HSPA1L*-T3	ATGATTTTCACCACCTACTCTG	TTAATCTACTTCTTCAATTG
*HSPA1L*-T4	ATGGCTACTGCCAAGGGAATCG	TTAAGCATCAAAAACAGTG
*HSPA1L*-T5	ATGAAACGTCTGATCGGCAGG	TTAATTGGTGACAGGGTGGC
*HSPA1L*-T6	ATGGCAGTGATTACCGTGCCAG	TTAATCTACTTCTTCAATTG
H5N1-*NA-*FL	ATGAATCCAAATCAAAAGATAA	TTACTTGTCAATGGTGAATGGC
H5N1-*NA-*N1	ATGAATCCAAATCAAAAGAT	TTATATTATGCCATTGTATTT
H5N1-*NA-*N2	ATGAGTATAAGGATTGGTTCCAA	TTAATTAGGGGACACCGGACCA
H5N1-*NA-*N3	ATGTGGCATGGTTCAAATCGGCC	TTACTTGTCAATGGTGAATGGCAA
H5N1-*NA-*N4	ATGACAGACACCATCAAG	TTACTGCCCATTACTTGGTCC
H5N1-*NA-*N5	ATGGCATCATATAAGATCTTC	TTAGATTTCGCCGGCATCAGG
H5N1-*NA*^K234R^	GGGCAGGCATCATATAGGATCTTCAAAATGG	CCATTTTGAAGATCCTATATGATGCCTGCCC
H5N1-*NA*^K240R^	CTTCAAAATGGAAAGAGGAAAAGTAATTAAATC	GATTTAATTACTTTTCCTCTTTCCATTTTGAAG
H5N1-*NA*^K242R^	CAAAATGGAAAAAGGAAGAGTAATTAAATCAGTC	GACTGATTTAATTACTCTTCCTTTTTCCATTTTG
H1N1-*NA*	ATGAATCCAAATCAGAAAATAATAA	CTACTTGTCAATGGTGAATGGC
H3N2-*NA*	GATGAATCCAAATCAAAAGATAATAA	TTATATAGGCATGAGATTGAGGTCC
H9N2-*NA*	ATGAATCCAAATCAGAAGATAA	TTATATAGGCATGAAGTTGATA
H7N9-*NA*	ATGAATCCAAATCAGAAGATTC	TTAGAGGAAGTACTCTATTTTAGC

### Quantitative real-time PCR analysis

Total RNA was extracted using Trizol reagent according to the manufacturer’s instructions. Subsequently, 1 µg of RNA was reverse-transcribed using the PrimeScript RT Reagent Kit with gDNA Eraser (RR047A, Takara). qPCR was performed with TB Green Premix Ex Taq (RR420A, Takara) on a LightCycler 96 system (Roche). *GAPDH* was used as an internal control to normalize the relative mRNA expression levels of *NA* gene. Data were analyzed using the 2^−ΔΔCt^ method and are presented as means ± SEM (*n* = 3 biological replicates). The following primers were used for qPCR:

*GAPDH* forward: 5ʹ- ATGACATCAAGAAGGTGGTG-3ʹ;

*GAPDH* reverse: 5ʹ- CATACCAGGAAATGAGCTTG-3ʹ;

*NA* forward: 5ʹ-ATTCATCTCATGCTCCCACT-3ʹ;

*NA* reverse: 5ʹ-CTGTGAGGGCTTCTGTCTTT-3ʹ.

### Protein expression and purification

The recombinant plasmids pGEX-4T-1-NA and pET-28a-HSPA1L were transformed into chemically competent *Escherichia coli* BL21 (pLysS) cells (Sangon Biotechnology, B528415-0010). Protein expression was induced by adding 1 mM isopropyl β-D-thiogalactopyranoside (IPTG, Sangon Biotechnology, A600168-0025) at 16°C with shaking at 90 rpm overnight. The pellets were harvested by centrifugation (4,000 × *g*, 10 min, 4°C) and subsequently resuspended in the respective binding buffers. Cell lysis was achieved through sonication on ice, followed by centrifugation at 12,000 × *g* for 10 min at 4°C to separate the soluble fraction. For His-HSPA1L purification, the lysate was incubated with Ni-NTA agarose (QIAGEN, 30210) in binding buffer containing 50 mM Tris-HCl (pH 8.0) and 10 mM imidazole. After extensive washing, the target protein was eluted with the same buffer containing 80 mM imidazole. For GST and GST-NA purification, the supernatant was mixed with GST Resin (GenScript, L00206) in binding buffer (50 mM Tris-HCl, pH 8.0, 150 mM NaCl), and the fusion proteins were eluted with 10 mg/5 mL reduced glutathione (Sangon Biotechnology, A100399-0005) in the same buffer.

### Mass spectrometry analysis

A549 cells infected with H5N1 virus (MOI = 3) for 18 h were collected and lysed using NP40 lysis buffer (P0013F; Beyotime, China) supplemented with PMSF (P0100; Solarbio, China). Cellular lysates were incubated with mouse anti-NA mAb or IgG as the control for 4 h, followed by incubation with protein A/G beads (sc-2003, Santa Cruz Biotechnology, CA, USA) for 4 h at 4°C. The immunoprecipitation complexes were separated by SDS-PAGE and stained with silver. Subsequently, the silver staining gels were manually excised and subjected to Liquid Chromatography Mass Spectrometry (LC-MS) analysis in APTBio (Shanghai, China).

### Viral infection and virus titer detection

A549 or HEK293T cells were infected with H5N1 (MOI = 3). At the indicated time points, the cells were collected and stored at −80℃. After three freeze-thaw cycles, the cellular lysates were centrifuged at 10,000 × *g* for 10 min at 4℃, and the supernatants were subjected to the 50% tissue culture infective dose (TCID_50_). Briefly, A549 cells grown on a 96-well plate were infected with IAV for 72 h. The cells were fixed with a methanol-acetone mixture (1:1, vol/vol) at −20°C for 20 min. The resultant cells were blocked with 5% skim milk diluted in phosphate-buffered saline (PBS) for 30 min at 37°C and incubated with mouse anti-M2 mAb for 2 h at 37°C, followed by incubation with FITC-conjugated goat anti-mouse IgG (172-1806, KPL) for 1.0 h at 37°C. Viral titers were determined by observing infected cells under a fluorescence microscope (OLYMPUS, U-RFL-T) and calculating the TCID_50_ per 0.1 mL.

### Confocal microscopy

To assess the colocalization of HSPA1L and H5N1-NA, A549 cells were seeded into 35 mm glass-bottom culture dishes and either mock-infected or infected with H5N1 the following day. At 12 h post-infection, the cells were fixed with 4% paraformaldehyde for 10 min and permeabilized with 0.2% Triton X-100 for 5 min at room temperature. After washing three times using PBS, the cells were incubated with anti-HSPA1L rabbit pAb and anti-NA mouse mAb overnight at 4℃. After washing three times with PBS, the cells were then incubated with FITC-labeled goat anti-mouse (172-1806, Kirkegaard & Perry Laboratories) and Alexa Fluor 568-conjugated goat anti-rabbit IgG H&L (A10040, Invitrogen) for 1 h at room temperature. Cellular nuclei were then stained with 4ʹ,6ʹ-diamidino-2-phenylindole (DAPI; 28718-90-3, BioFroxx) for 10 min at room temperature. Images were acquired using an LSM780 laser scanning confocal microscope (Zeiss, Germany).

### Co-IP and GST pull-down assays

For co-IP assays, cells were lysed in NP-40 lysis buffer (Beyotime, P0013F; Shanghai, China) supplemented with 1 mM phenylmethylsulfonyl fluoride (PMSF) for 4 h or overnight at 4°C. After centrifugation, the supernatant was incubated with Flag beads, Myc beads, or specific antibodies coupled to protein A/G PLUS-agarose at 4°C for 4 h or overnight.

For GST pull-down assays, purified GST and GST-NA proteins were individually incubated with His-HSPA1L at 4°C for 4 h. Subsequently, GST resin (60 µL) was added to capture the protein complexes. After incubation, the resin was pelleted by centrifugation, washed five times with NP-40 buffer at 4°C to remove non-specific interactions, and then lysed for immunoblotting analysis.

### IAV rescue

Recombinant viruses carrying WT NA or the NA^K242R^ mutation (designated rWT and rK242R, respectively) were generated using reverse genetics as previously described (55). Briefly, the plasmid pLLBA-H5N1-NA^WT^ or pLLBA-H5N1-NA^K242R^ was co-transfected into HEK293T cells alongside the following plasmids: pLLBA-H5N1-PA, pLLBA-H5N1-PB2, pLLBA-H5N1-PB1, pLLBA-H5N1-NP, pLLBA-H5N1-HA, pLLBA-H5N1-NS, and pLLBA-H5N1-M. At 24 h post-transfection, the cells were treated with 1 µg/mL TPCK-treated trypsin (Worthington Biochemicals, LS003750) for 36 h to facilitate viral propagation. The recombinant viruses (rWT and rK242R) were then individually inoculated into 9-day-old specific pathogen-free (SPF) embryonated chicken eggs and allowed to propagate for 3 days. The viruses were subsequently harvested and stored at −80°C until further use.

### Western blot analysis

Protein samples were prepared using RIPA buffer (Beyotime, P0013C) and separated by SDS-PAGE. The separated protein bands were then transferred to nitrocellulose membranes (LABSELECT, TM-NC-R-45). The membranes were blocked with 5% skimmed milk in PBS containing 0.1% Tween 20 (PBST) at room temperature. After blocking, the membranes were washed three times with PBST and incubated with the indicated primary antibodies overnight at 4°C. Following five additional washes with PBST, the membranes were incubated with HRP-conjugated secondary antibodies for 1 h at room temperature. After a final wash (three to five times), the protein bands were visualized using Superkine Chemiluminescence Substrate (Abbkine, BMU102-CN) and imaged with the GelView 9000 Lite system (Biolight Biotechnology Co., Ltd). β-actin expression was used as a loading control for normalization. Protein bands were quantified by Image J software and shown as mean ± SEM (*n* = 3 biological replicates).

### CRISPR-Cas9 knockout

The *HSPA1L* gene target sequence (5′- AATCGCCATAGGCATCGACC-3′), *NBR1* gene target sequence (5′- CTGATCCAGAAAATACAACT-3′), and *TAX1BP1* gene target sequence (5′-TTACCTTCCTAATGCACACC-3′) were separately cloned into the guide RNA expression plasmid Lenti-CRISPR-V2 (Addgene, 52961, depositing lab: Feng Zhang from Broad Institute). The resultant recombinant lentiviral plasmids, along with psPAX2 (Addgene, 12260) and pMD2.G (Addgene, 12259) packaging plasmids, were co-transfected into HEK293KT cells using Polyethylenimine HCl MAX, Linear (P4000, LABLEAD) for 48 h. After centrifugation at 10,000 × *g* for 10 min at 4°C, the supernatants were added to HEK293T cells. At 24 h after infection, the resultant cells were selected with puromycin (5 µg/mL; Invivogen, 58-58-2) for 72 h. Finally, monoclonal knockout cell strains were obtained by the limiting dilution method. Immunoblotting assays were used to identify knockout cell lines.

### Cell viability assays

Cell viability was assessed using a Cell Counting Kit-8 (CCK-8; C0037, Beyotime) following the manufacturer’s protocol. To evaluate the effects of drug pretreatment, cells were seeded in 96-well plates at a density of 1 × 10³ cells/mL per well and exposed to the following compounds at graded concentrations: DMSO (control), MG132 (2.5, 5, 10, and 20 µM), BafA1 (25, 50, 100, and 200 nM), Wort (5, 10, 20, and 40 µM), CHX (25, 50, 100, and 200 µg/mL). After 6 h of treatment, 10 µL of CCK-8 solution was added to each well, followed by a 2 h incubation. Absorbance was measured at 450 nm using a microplate reader.

### Statistical analysis

The data were analyzed using GraphPad Prism software version 9.0 by unpaired Student’s *t*-test or two-way ANOVA (*P < 0.05, **P < 0.01, ***P < 0.001, not significant (ns): *P* > 0.05). Each experiment was independently performed with three biological repeats. All results are presented as mean ± SEM.

## Data Availability

All data from this study are included in the paper and are available from the corresponding author upon reasonable request.

## References

[1] Horimoto T, Kawaoka Y. 2005. Influenza: lessons from past pandemics, warnings from current incidents. Nat Rev Microbiol 3:591–600. doi:10.1038/nrmicro1208 16064053

[2] Charostad J, Rezaei Zadeh Rukerd M, Mahmoudvand S, Bashash D, Hashemi SMA, Nakhaie M, Zandi K. 2023. A comprehensive review of highly pathogenic avian influenza (HPAI) H5N1: an imminent threat at doorstep. Travel Med Infect Dis 55:102638. doi:10.1016/j.tmaid.2023.102638 37652253

[3] Eisfeld AJ, Biswas A, Guan L, Gu C, Maemura T, Trifkovic S, Wang T, Babujee L, Dahn R, Halfmann PJ, Barnhardt T, Neumann G, Suzuki Y, Thompson A, Swinford AK, Dimitrov KM, Poulsen K, Kawaoka Y. 2024. Pathogenicity and transmissibility of bovine H5N1 influenza virus. Nature 633:426–432. doi:10.1038/s41586-024-07766-6 38977017 PMC11390473

[4] Burrough ER, Magstadt DR, Petersen B, Timmermans SJ, Gauger PC, Zhang J, Siepker C, Mainenti M, Li G, Thompson AC, Gorden PJ, Plummer PJ, Main R. 2024. Highly Pathogenic Avian Influenza A(H5N1) Clade 2.3.4.4b Virus Infection in Domestic Dairy Cattle and Cats, United States, 2024. Emerg Infect Dis 30:1335–1343. doi:10.3201/eid3007.240508 38683888 PMC11210653

[5] Hu Y, Chen X, Ling Y, Zhou K, Han M, Wang X, Yue M, Li Y. 2023. Influenza A virus inhibits TET2 expression by endoribonuclease PA-X to attenuate type I interferon signaling and promote viral replication. PLoS Pathog 19:e1011550. doi:10.1371/journal.ppat.1011550 37498975 PMC10409264

[6] Reich S, Guilligay D, Pflug A, Malet H, Berger I, Crépin T, Hart D, Lunardi T, Nanao M, Ruigrok RWH, Cusack S. 2014. Structural insight into cap-snatching and RNA synthesis by influenza polymerase. Nature 516:361–366. doi:10.1038/nature14009 25409151

[7] Te Velthuis AJW, Grimes JM, Fodor E. 2021. Structural insights into RNA polymerases of negative-sense RNA viruses. Nat Rev Microbiol 19:303–318. doi:10.1038/s41579-020-00501-8 33495561 PMC7832423

[8] Chauhan RP, Gordon ML. 2022. An overview of influenza A virus genes, protein functions, and replication cycle highlighting important updates. Virus Genes 58:255–269. doi:10.1007/s11262-022-01904-w 35471490

[9] Wagner R, Matrosovich M, Klenk HD. 2002. Functional balance between haemagglutinin and neuraminidase in influenza virus infections. Rev Med Virol 12:159–166. doi:10.1002/rmv.352 11987141

[10] Li Y, Cao H, Dao N, Luo Z, Yu H, Chen Y, Xing Z, Baumgarth N, Cardona C, Chen X. 2011. High-throughput neuraminidase substrate specificity study of human and avian influenza A viruses. Virology (Auckl) 415:12–19. doi:10.1016/j.virol.2011.03.024 PMC3114948 21501853

[11] Gaur P, Ranjan P, Sharma S, Patel JR, Bowzard JB, Rahman SK, Kumari R, Gangappa S, Katz JM, Cox NJ, Lal RB, Sambhara S, Lal SK. 2012. Influenza A virus neuraminidase protein enhances cell survival through interaction with carcinoembryonic antigen-related cell adhesion molecule 6 (CEACAM6) protein. Journal of Biological Chemistry 287:15109–15117. doi:10.1074/jbc.M111.328070 22396546 PMC3340274

[12] Kumar P, Gaur P, Kumari R, Lal SK. 2019. Influenza A virus neuraminidase protein interacts with Hsp90, to stabilize itself and enhance cell survival. J Cell Biochem 120:6449–6458. doi:10.1002/jcb.27935 30335904

[13] Webster RG, Air GM, Metzger DW, Colman PM, Varghese JN, Baker AT, Laver WG. 1987. Antigenic structure and variation in an influenza virus N9 neuraminidase. J Virol 61:2910–2916. doi:10.1128/JVI.61.9.2910-2916.1987 3612957 PMC255818

[14] Colman PM, Varghese JN, Laver WG. 1983. Structure of the catalytic and antigenic sites in influenza virus neuraminidase. Nature 303:41–44. doi:10.1038/303041a0 6188957

[15] Yong J, Lu S, Lu C, Huang R. 2025. The development history, structural composition, and functions of influenza viruses and the progress of influenza virus inhibitors in clinics and clinical trials. Mini Rev Med Chem 25:196–207. doi:10.2174/0113895575316416240724043949 39113298

[16] Wu KW, Chien CY, Li SW, King CC, Chang CH. 2012. Highly conserved influenza A virus epitope sequences as candidates of H3N2 flu vaccine targets. Genomics 100:102–109. doi:10.1016/j.ygeno.2012.06.003 22698979

[17] Hagymasi AT, Dempsey JP, Srivastava PK. 2022. Heat-shock proteins. Curr Protoc 2:e592. doi:10.1002/cpz1.592 36367390

[18] Zhang X, Yu W. 2022. Heat shock proteins and viral infection. Front Immunol 13:947789. doi:10.3389/fimmu.2022.947789 35990630 PMC9389079

[19] Rosenzweig R, Nillegoda NB, Mayer MP, Bukau B. 2019. The Hsp70 chaperone network. Nat Rev Mol Cell Biol 20:665–680. doi:10.1038/s41580-019-0133-3 31253954

[20] Wu HH, Wang B, Armstrong SR, Abuetabh Y, Leng S, Roa WHY, Atfi A, Marchese A, Wilson B, Sergi C, Flores ER, Eisenstat DD, Leng RP. 2021. Hsp70 acts as a fine-switch that controls E3 ligase CHIP-mediated TAp63 and ΔNp63 ubiquitination and degradation. Nucleic Acids Res 49:2740–2758. doi:10.1093/nar/gkab081 33619536 PMC7969027

[21] Flaherty KM, DeLuca-Flaherty C, McKay DB. 1990. Three-dimensional structure of the ATPase fragment of a 70K heat-shock cognate protein. Nature 346:623–628. doi:10.1038/346623a0 2143562

[22] Daugaard M, Rohde M, Jäättelä M. 2007. The heat shock protein 70 family: highly homologous proteins with overlapping and distinct functions. FEBS Lett 581:3702–3710. doi:10.1016/j.febslet.2007.05.039 17544402

[23] Radons J. 2016. The human HSP70 family of chaperones: where do we stand? Cell Stress Chaperones 21:379–404. doi:10.1007/s12192-016-0676-6 26865365 PMC4837186

[24] Zuiderweg ERP, Hightower LE, Gestwicki JE. 2017. The remarkable multivalency of the Hsp70 chaperones. Cell Stress Chaperones 22:173–189. doi:10.1007/s12192-017-0776-y 28220454 PMC5352603

[25] Wisniewska M, Karlberg T, Lehtio L, Johansson I, Kotenyova T, Moche M, Schuler H. 2010. Crystal structures of the ATPase domains of four human Hsp70 isoforms: HSPA1L/Hsp70-hom, HSPA2/Hsp70-2, HSPA6/Hsp70B’, and HSPA5/BiP/GRP78. PLoS ONE:e8625. doi:10.1371/journal.pone.0008625 20072699 PMC2803158

[26] Hou L, Zeng P, Wu Z, Yang X, Guo J, Shi Y, Song J, Zhou J, Liu J. 2024. Heat shock protein 70 enhances viral replication by stabilizing Senecavirus A nonstructural proteins L and 3D. Vet Res 55:158. doi:10.1186/s13567-024-01414-7 39695881 PMC11654094

[27] Taguwa S, Yeh MT, Rainbolt TK, Nayak A, Shao H, Gestwicki JE, Andino R, Frydman J. 2019. Zika virus dependence on host Hsp70 provides a protective strategy against infection and disease. Cell Rep 26:906–920. doi:10.1016/j.celrep.2018.12.095 30673613 PMC6709865

[28] Xu T, Lin Z, Wang C, Li Y, Xia Y, Zhao M, Hua L, Chen Y, Guo M, Zhu B. 2019. Heat shock protein 70 as a supplementary receptor facilitates enterovirus 71 infections in vitro. Microb Pathog 128:106–111. doi:10.1016/j.micpath.2018.12.032 30579945

[29] Alam SB, Rochon D. 2015. Cucumber necrosis virus recruits cellular heat shock protein 70 homologs at several stages of infection. J Virol 90:3302–3317. doi:10.1128/JVI.02833-15 26719261 PMC4794660

[30] Dong S, Liu L, Wu W, Armstrong SD, Xia D, Nan H, Hiscox JA, Chen H. 2016. Determination of the interactome of non-structural protein12 from highly pathogenic porcine reproductive and respiratory syndrome virus with host cellular proteins using high throughput proteomics and identification of HSP70 as a cellular factor for virus replication. J Proteomics 146:58–69. doi:10.1016/j.jprot.2016.06.019 27327135

[31] Cao J, Liu S, Liu M, Wang S, Bi Z, Fan W, Shi Z, Song S, Yan L. 2022. Hsp70 inhibits the replication of fowl adenovirus serotype 4 by suppressing viral hexon with the assistance of DnaJC7. J Virol 96:e0080722. doi:10.1128/jvi.00807-22 35852354 PMC9364783

[32] Li G, Zhang J, Tong X, Liu W, Ye X. 2011. Heat shock protein 70 inhibits the activity of Influenza A virus ribonucleoprotein and blocks the replication of virus in vitro and in vivo. PLoS One 6:e16546. doi:10.1371/journal.pone.0016546 21390211 PMC3044721

[33] Kim MY, Shu Y, Carsillo T, Zhang J, Yu L, Peterson C, Longhi S, Girod S, Niewiesk S, Oglesbee M. 2013. Hsp70 and a novel axis of type I interferon-dependent antiviral immunity in the measles virus-infected brain. J Virol 87:998–1009. doi:10.1128/JVI.02710-12 23135720 PMC3554074

[34] Pujhari S, Brustolin M, Macias VM, Nissly RH, Nomura M, Kuchipudi SV, Rasgon JL. 2019. Heat shock protein 70 (Hsp70) mediates Zika virus entry, replication, and egress from host cells. Emerg Microbes Infect 8:8–16. doi:10.1080/22221751.2018.1557988 30866755 PMC6455116

[35] Hou G, Zhang Q, Li C, Ding G, Hu L, Chen X, Lv Z, Fan Y, Zou J, Xiao T, Zhang YA, Li J. 2023. An aquareovirus exploits membrane-anchored HSP70 to promote viral entry. Microbiol Spectr 11:e0405522. doi:10.1128/spectrum.04055-22 37158746 PMC10269764

[36] Guo J, Yan Y, Sun J, Ji K, Hei Z, Zeng L, Xu H, Ren X, Sun Y. 2024. Chaperones Hsc70 and Hsp70 play distinct roles in the replication of bocaparvovirus minute virus of canines. Mol Microbiol 121:1127–1147. doi:10.1111/mmi.15263 38629786

[37] Khachatoorian R, Riahi R, Ganapathy E, Shao H, Wheatley NM, Sundberg C, Jung CL, Ruchala P, Dasgupta A, Arumugaswami V, Gestwicki JE, French SW. 2016. Allosteric heat shock protein 70 inhibitors block hepatitis C virus assembly. Int J Antimicrob Agents 47:289–296. doi:10.1016/j.ijantimicag.2016.01.012 27013001 PMC4833571

[38] Sargent CA, Dunham I, Trowsdale J, Campbell RD. 1989. Human major histocompatibility complex contains genes for the major heat shock protein HSP70. Proc Natl Acad Sci USA 86:1968–1972. doi:10.1073/pnas.86.6.1968 2538825 PMC286826

[39] Milner CM, Campbell RD. 1990. Structure and expression of the three MHC-linked HSP70 genes. Immunogenetics 32:242–251. doi:10.1007/BF00187095 1700760

[40] da Silva DV, Nordholm J, Dou D, Wang H, Rossman JS, Daniels R. 2015. The influenza virus neuraminidase protein transmembrane and head domains have coevolved. J Virol 89:1094–1104. doi:10.1128/JVI.02005-14 25378494 PMC4300628

[41] Nordholm J, da Silva DV, Damjanovic J, Dou D, Daniels R. 2013. Polar residues and their positional context dictate the transmembrane domain interactions of influenza A neuraminidases. J Biol Chem 288:10652–10660. doi:10.1074/jbc.M112.440230 23447533 PMC3624445

[42] Ferretti GDS, Quaas CE, Bertolini I, Zuccotti A, Saatci O, Kashatus JA, Sharmin S, Lu DY, Poli ANR, Quesnelle AF, Rodriguez-Blanco J, de Cubas AA, Hobbs GA, Liu Q, O’Bryan JP, Salvino JM, Kashatus DF, Sahin O, Barnoud T. 2024. HSP70-mediated mitochondrial dynamics and autophagy represent a novel vulnerability in pancreatic cancer. Cell Death Differ 31:881–896. doi:10.1038/s41418-024-01310-9 38802657 PMC11239841

[43] Wang B, Chen Z, Yu F, Chen Q, Tian Y, Ma S, Wang T, Liu X. 2016. Hsp90 regulates autophagy and plays a role in cancer therapy. Tumour Biol 37:1–6. doi:10.1007/s13277-015-4142-3 26432328

[44] Deng T, Hu B, Wang X, Ding S, Lin L, Yan Y, Peng X, Zheng X, Liao M, Jin Y, Dong W, Gu J, Zhou J. 2022. TRAF6 autophagic degradation by avibirnavirus VP3 inhibits antiviral innate immunity via blocking NFKB/NF-κB activation. Autophagy 18:2781–2798. doi:10.1080/15548627.2022.2047384 35266845 PMC9673932

[45] Han JH, Jang KW, Myung CS. 2022. Garcinia cambogia attenuates adipogenesis by affecting CEBPB and SQSTM1/p62-mediated selective autophagic degradation of KLF3 through RPS6KA1 and STAT3 suppression. Autophagy 18:518–539. doi:10.1080/15548627.2021.1936356 34101546 PMC9037513

[46] Wang T, Luo R, Zhang J, Lu Z, Li LF, Zheng YH, Pan L, Lan J, Zhai H, Huang S, Sun Y, Qiu HJ. 2023. The MGF300-2R protein of African swine fever virus is associated with viral pathogenicity by promoting the autophagic degradation of IKKα and IKKβ through the recruitment of TOLLIP. PLoS Pathog 19:e1011580. doi:10.1371/journal.ppat.1011580 37566637 PMC10446188

[47] Li K, Chen D, Zhao K, Liu D, Kong D, Sun Y, Guan A, Zhou P, Jin H, Jongkaewwattana A, Suolang S, Wang D, Zhou H, Luo R. 2025. Cleavage of the selective autophagy receptor NBR1 by the PDCoV main protease NSP5 impairs autophagic degradation of the viral envelope protein. Autophagy. doi:10.1080/15548627.2025.2474576:1-16 PMC12283008 40047225

[48] Zeng Y, Xu S, Wei Y, Zhang X, Wang Q, Jia Y, Wang W, Han L, Chen Z, Wang Z, Zhang B, Chen H, Lei CQ, Zhu Q. 2021. The PB1 protein of influenza A virus inhibits the innate immune response by targeting MAVS for NBR1-mediated selective autophagic degradation. PLoS Pathog 17:e1009300. doi:10.1371/journal.ppat.1009300 33577621 PMC7880438

[49] Campbell LK, Magor KE. 2020. Pattern recognition receptor signaling and innate responses to influenza A viruses in the mallard duck, compared to humans and chickens. Front Cell Infect Microbiol 10:209. doi:10.3389/fcimb.2020.00209 32477965 PMC7236763

[50] Gack MU, Albrecht RA, Urano T, Inn K-S, Huang I-C, Carnero E, Farzan M, Inoue S, Jung JU, García-Sastre A. 2009. Influenza A virus NS1 targets the ubiquitin ligase TRIM25 to evade recognition by the host viral RNA sensor RIG-I. Cell Host Microbe 5:439–449. doi:10.1016/j.chom.2009.04.006 19454348 PMC2737813

[51] Choudhury NR, Trus I, Heikel G, Wolczyk M, Szymanski J, Bolembach A, Dos Santos Pinto RM, Smith N, Trubitsyna M, Gaunt E, Digard P, Michlewski G. 2022. TRIM25 inhibits influenza A virus infection, destabilizes viral mRNA, but is redundant for activating the RIG-I pathway. Nucleic Acids Res 50:7097–7114. doi:10.1093/nar/gkac512 35736141 PMC9262604

[52] Lubkowska A, Pluta W, Strońska A, Lalko A. 2021. Role of heat shock proteins (HSP70 and HSP90) in viral infection. Int J Mol Sci 22:9366. doi:10.3390/ijms22179366 34502274 PMC8430838

[53] Chen N, Liu Y, Bai T, Chen J, Zhao Z, Li J, Shao B, Zhang Z, Zhou Y, Wang X, Zhu Z. 2022. Quercetin inhibits Hsp70 blocking of bovine viral diarrhea virus infection and replication in the early stage of virus infection. Viruses 14:2365. doi:10.3390/v14112365 36366463 PMC9692758

[54] Jiao Y, Zhao P, Xu LD, Yu JQ, Cai HL, Zhang C, Tong C, Yang YL, Xu P, Sun Q, Chen N, Wang B, Huang YW. 2024. Enteric coronavirus nsp2 is a virulence determinant that recruits NBR1 for autophagic targeting of TBK1 to diminish the innate immune response. Autophagy 20:1762–1779. doi:10.1080/15548627.2024.2340420 38597182 PMC11262224

[55] Lin L, Wang X, Chen Z, Deng T, Yan Y, Dong W, Huang Y, Zhou J. 2023. TRIM21 restricts influenza A virus replication by ubiquitination-dependent degradation of M1. PLoS Pathog 19:e1011472. doi:10.1371/journal.ppat.1011472 37343022 PMC10325077

[56] Sun N, Jiang L, Ye M, Wang Y, Wang G, Wan X, Zhao Y, Wen X, Liang L, Ma S, Liu L, Bu Z, Chen H, Li C. 2020. TRIM35 mediates protection against influenza infection by activating TRAF3 and degrading viral PB2. Protein Cell 11:894–914. doi:10.1007/s13238-020-00734-6 32562145 PMC7719147

[57] Wang Z, Chen J, Wu X, Ma D, Zhang X, Li R, Han C, Liu H, Yin X, Du Q, Tong D, Huang Y. 2021. PCV2 targets cGAS to inhibit type I interferon induction to promote other DNA virus infection. PLoS Pathog 17:e1009940. doi:10.1371/journal.ppat.1009940 34543359 PMC8483418

[58] Xie W, Tian S, Yang J, Cai S, Jin S, Zhou T, Wu Y, Chen Z, Ji Y, Cui J. 2022. OTUD7B deubiquitinates SQSTM1/p62 and promotes IRF3 degradation to regulate antiviral immunity. Autophagy 18:2288–2302. doi:10.1080/15548627.2022.2026098 35100065 PMC9542415

[59] Jin S, Tian S, Luo M, Xie W, Liu T, Duan T, Wu Y, Cui J. 2017. Tetherin suppresses type i interferon signaling by targeting MAVS for NDP52-mediated selective autophagic degradation in human cells. Mol Cell 68:308–322. doi:10.1016/j.molcel.2017.09.005 28965816

[60] Di Pietro A, Kajaste-Rudnitski A, Oteiza A, Nicora L, Towers GJ, Mechti N, Vicenzi E. 2013. TRIM22 inhibits influenza A virus infection by targeting the viral nucleoprotein for degradation. J Virol 87:4523–4533. doi:10.1128/JVI.02548-12 23408607 PMC3624352

[61] Fu B, Wang L, Ding H, Schwamborn JC, Li S, Dorf ME. 2015. TRIM32 senses and restricts influenza A virus by ubiquitination of PB1 polymerase. PLoS Pathog 11:e1004960. doi:10.1371/journal.ppat.1004960 26057645 PMC4461266

[62] Song W, Wang P, Mok BW-Y, Lau S-Y, Huang X, Wu W-L, Zheng M, Wen X, Yang S, Chen Y, Li L, Yuen K-Y, Chen H. 2014. The K526R substitution in viral protein PB2 enhances the effects of E627K on influenza virus replication. Nat Commun 5:5509. doi:10.1038/ncomms6509 25409547 PMC4263149

[63] Mehle A, Doudna JA. 2009. Adaptive strategies of the influenza virus polymerase for replication in humans. Proc Natl Acad Sci USA 106:21312–21316. doi:10.1073/pnas.0911915106 19995968 PMC2789757

[64] Sun X, Belser JA, Maines TR. 2020. Adaptation of H9N2 influenza viruses to mammalian hosts: a review of molecular markers. Viruses 12:541. doi:10.3390/v12050541 32423002 PMC7290818

[65] Gabriel G, Dauber B, Wolff T, Planz O, Klenk HD, Stech J. 2005. The viral polymerase mediates adaptation of an avian influenza virus to a mammalian host. Proc Natl Acad Sci USA 102:18590–18595. doi:10.1073/pnas.0507415102 16339318 PMC1317936

[66] Sutton TC, Finch C, Shao H, Angel M, Chen H, Capua I, Cattoli G, Monne I, Perez DR. 2014. Airborne transmission of highly pathogenic H7N1 influenza virus in ferrets. J Virol 88:6623–6635. doi:10.1128/JVI.02765-13 24696487 PMC4054360

[67] Bi Z, Ye H, Wang X, Fang A, Yu T, Yan L, Zhou J. 2019. Insights into species-specific regulation of ANP32A on the mammalian-restricted influenza virus polymerase activity. Emerg Microbes Infect 8:1465–1478. doi:10.1080/22221751.2019.1676625 31608791 PMC6818127

[68] Lutz M, Schmierer J, Takimoto T. 2022. Host adaptive mutations in the 2009 H1N1 pandemic influenza A virus PA gene regulate translation efficiency of viral mRNAs via GRSF1. Commun Biol 5:1102. doi:10.1038/s42003-022-04082-5 36253464 PMC9576711

